# High Center‐of‐Mass, Multi‐Legged Soft Robots Powered by Geometrically Encoded Liquid Crystal Elastomer Arc Appendages

**DOI:** 10.1002/adma.73794

**Published:** 2026-06-19

**Authors:** Jong Bin Kim, Antonio Proctor Martinez, Yaoye Hong, Ziyun Zhang, Kun‐Yu Wang, Shu Yang

**Affiliations:** ^1^ Department of Materials Science and Engineering University of Pennsylvania Philadelphia Pennsylvania USA

**Keywords:** arc appendages, fibers, high center‐of‐mass, liquid crystal elastomers, soft robots

## Abstract

Biological appendages are paramount for locomotion by combining high compliance and versatile maneuverability. Yet most soft robots are largely confined to the contact surface due to their appendage‐free and single‐mode deformation, resulting in a low center‐of‐mass (CoM) and limited postural/spatial mobility. Here, we create soft robots utilizing liquid crystal elastomer (LCE) arc fibers as appendages to emulate the complex maneuvers of their biological counterparts. Geometrically encoding combined torsional and flexural modes allows these robots to transcend surface constraints via a naturally elevated CoM and enhanced maneuverability. Leveraging the kinetics and thermodynamics of the fiber deformation‐recovery cycles, we integrate the encoded fibers into the 3D‐printed body with vertical and horizontal rotational symmetries, inspired by the locomotion of the octopus and the golden wheel spider, respectively. Our model can elevate, lower, tilt, and rotate with substantial postural freedom. It can also be engineered to roll at 1.3 body lengths per second, climb a 32.5° incline, and traverse unstructured terrain. These feats are enabled by the large inertia generated by the inherent instability of the raised CoM and the effective ground anchoring of the appendages. These insights lay the foundation for customizable, high‐mobility soft robotic platforms that navigate complex real‐world environments.

## Graphical abstract

Inspired by the octopus and the golden wheel spider, soft robots with liquid crystal elastomer arc fibers as appendages are fabricated to transcend surface constraints through an elevated center of mass and minimal contact footprints. By leveraging curvature‐encoded deformation‐recovery cycles, these robots exhibit contractile, torsional, and flexural modes, enabling exceptional postural freedom and agile, autonomous locomotion across complex terrain.

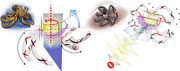

## 1 Introduction

Organisms can achieve compliant, complex postural changes and whole‐body movements on diverse surfaces using their appendages. For example, an octopus extends, twists, and bends its arms, enabling highly versatile body movement [1]. The golden wheel spider employs its legs not only for walking but also for rolling sideways down slopes to escape predators [2]. In these examples, the exceptional freedom of movement is primarily driven by the appendages' geometric configurations and complex deformations. Such structures naturally result in a raised center‐of‐mass (CoM), enabling whole‐body maneuverability with minimal contact with the ground. The dynamic superiority and adaptability demonstrated by these natural appendages provide inspiration for creating robots [3] that can adapt to complex, unstructured terrain [4].

In soft robotics, appendages are often realized through ground‐contacting sheets, rods, or fibers actuated by an electromagnetic field‐induced Lorentz force on charged particles [5], electrostatic pressure applied to dielectric elastomer actuators (DEAs) under ultrahigh voltage [6], or thermomechanical deformation of shape memory polymers [7]. They inherently have low maneuverability due to their monolithic, planar nature and/or linear contact with the ground. Although diverse morphologies can be generated by patterning the body and tuning the external stimuli [8, 9, 10, 11], the locomotion of appendages typically relies on asymmetrical friction forces in contact with the substrate [5, 10, 11]. While multi‐component DEAs at junctions allow multi‐directional bending of appendages, complex deformations within the appendage itself remain rarely demonstrated [8, 9]. Considering alternative appendage materials, while ionic polymer‐metal composites are hindered by slow response speeds and the need for a hydrated environment [12], fluidic actuators typically maintain a restricted, low‐profile body geometry despite their rapid actuation [13].

Liquid crystal elastomers (LCEs), which are lightly cross‐linked polymer networks of mesogens, are well‐suited for enabling large, complex, and reversible deformations [14, 15, 16, 17]. LCEs undergo macroscopic shape changes in response to temperature, driven by a phase transition of the mesogens from an aligned state (e.g., the nematic phase) to an isotropic state. Although strips or fibers with mere contractile deformation achieve locomotion through a specific setup [18, 19, 20], they lack the capacity for complex shape‐morphing. More complex alignment and deformation are enabled by employing mechanical [21], surface‐enforced [22, 23, 24, 25, 26], extrusion‐based shear [27, 28, 29], or magnetic‐field‐assisted alignment [30], or by adopting bilayer films [31, 32] or Janus/core–shell fibers [33, 34]. In addition, structurally transforming planar films or linear fibers into twisted or bent geometries is a critical step in programming the mechanical responses of LCE architectures. While many twisted fibers demonstrate rectilinear rotation [35, 36] or off‐axis/non‐linear motion [37, 38] when supporting a dangling load, twist remains a primary driving force, either for independent rolling on a hot plate or in combination with specific mechanical setups [37, 39]. Coupling specific geometries—such as bent strips [40], twisted strips [41, 42, 43, 44, 45, 46, 47, 48], coils [39, 49], fibers/strips straightened from a bent state [38, 43, 50], and ring topologies [51, 52, 53, 54, 55]—leverages structural asymmetry and pre‐strained states to convert simple molecular contractions into more complex out‐of‐plane motions. A wheel made from LCE films achieves autonomous and continuous rolling locomotion [48, 56]. Nevertheless, these soft robots are appendage‐free and rely on single‐mode deformations. This leads to a planar or macroscopically one‐dimensional geometry with a low CoM, which inherently restricts vertical movement and narrows the design space of locomotion modes. While versatile locomotion modes have been demonstrated in LCE kirigami films by the design of defect arrays [57], mimicking octopus appendages that combine elongational, torsional, and flexural modes with a minimal contact footprint remains challenging.

Here, we create soft robots with multiple appendages that are made from LCE semicircular arc fibers. We specifically adopt this curved geometry because its inherent curvature elevates the robot's body and provides an ideal initial state for point contact, while encoding the mechanical constraints needed to translate thermal stimuli into simultaneous contractile, torsional, and flexural deformations, much like an octopus's arms. By programming locomotion through each appendage's deformation, we investigate the dynamic interplay between arc geometry (curvature, *κ*) and mesogen alignment. Upon heating, instead of being straightened, LCE arcs bend further as the initial *κ* decreases or the mesogen‐to‐chain‐extender ratio increases; conversely, they unbend toward the opposite curvature when the conditions are reversed. These behaviors are inferred from the alignment contrast shown in X‐ray diffraction (XRD) patterns. This suggests that the migration of dangling chains in the precursors during bending leads to a disparity in crosslinking density between the inner and outer arcs, which is validated by atomic force microscopy (AFM) indentation tests. Integrating the programmed appendages along a circular frame raises the CoM, allowing for complex movements like those seen in an octopus and a golden wheel spider, including postural changes and rolling locomotion. The octopus model can rise to 338% of its initial body height, lower to 50%, rotate by 60°, and tilt to 39.4°. The spider model can roll up to 1.3 body lengths per second and adapt to shallow water, climb a 32.5° incline, or roll on unstructured, crumpled terrain. Importantly, the diverse motions are enabled by simple actuation inputs acting on geometrically optimized appendages, achieving a level of agility unattainable in conventional appendage‐free, low‐CoM soft robots.

## 2 Results and Discussions

### 2.1 Fabrication of the LCE Arc Fibers and Overall Robot Designs

**FIGURE 1:**
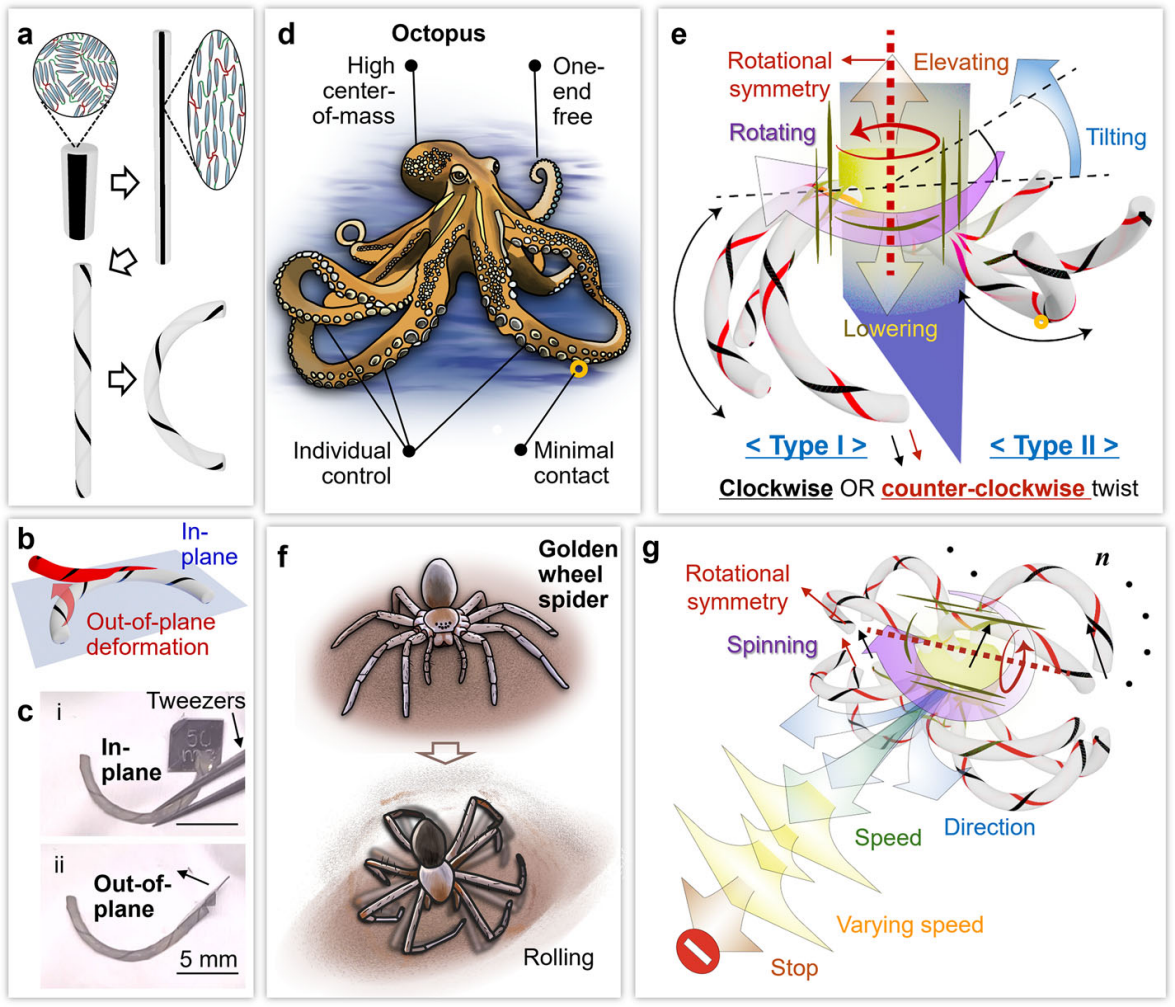
Design of the bioinspired soft robots with multi‐appendages made from the LCE fibers. (a) Illustration of the two‐step crosslinking process to simultaneously twist and bend the LCE fibers. (b) Illustration of the out‐of‐plane deformation of the fiber upon heating. (c) Optical images of an LCE fiber with a steel plate attached to one end on a hot plate at 180°C, demonstrating the flipping of the plate and thus the out‐of‐plane deformation of the fiber. (d) Illustration of an octopus with advantageous features for soft robots. (e) Illustrations of an octopus‐inspired soft robot design with a vertical rotational symmetry, which can elevate, lower, rotate, or tilt the body, respectively. The distinction between Type I and Type II is determined by the angle of fiber attachment to the body. (f) Illustration of a golden wheel spider that rolls with the help of its legs. (g) Illustrations of a golden wheel spider‐inspired soft robot design with horizontal rotational symmetry, which can roll with multiple variations and spin in place.

For the appendages of the soft robot, the semicircular geometry is adopted because it provides an ideal initial state for point contact with the substrate and elevates the center of mass (CoM) through its inherent curvature. This structure is uniquely suited for simultaneously imposing tensile, torsional, and flexural deformations, effectively mimicking the complex deformations observed in an octopus's arms. The semicircular LCE arc fibers are fabricated in three stages: extension and partial crosslinking of the mesogens via Michael addition reactions (Stage I), mechanical alignment by stretching, twisting, and bending (Stage II), and photo‐crosslinking (Stage III) (see Figure 1a and Experimental Section/Methods) [21]. At Stage I, the acrylate groups of the mesogens thermally react with the thiol groups of the chain extenders and crosslinkers, assisted by a catalyst. The stoichiometric ratio (*R*), defined as the molar ratio of *n*(acrylate)/*n*(thiol), is controlled to be slightly greater than 1; the resulting fibers are hereafter referred to as *R*1.1 (*R* = 1.1) and *R*1.25 (*R* = 1.25), respectively. The excess acrylate groups, referred to as dangling chains, are utilized in Stage III to lock the encoded alignment via UV crosslinking. At Stage II, the partially crosslinked fibers are stretched, twisted, and bent concurrently to encode the deformation modes seen in octopus's arms.

Upon heating above the nematic‐to‐isotropic phase transition temperature (*T*_NI_), the LCE fiber is expected to shrink anisotropically along the alignment direction. Therefore, the twisted and bent LCE fibers, cut into semicircular arcs, undergo both torsional and flexural deformation, resulting in out‐of‐plane actuation (Figure 1b), as evidenced by the flipping of a steel plate attached to one end of the fiber (Figure 1c, Figure S1 and Movie S1). The fibers can be chemomechanically encoded by several factors: *R*, twist density, twist handedness, *κ*, and fiber diameter (Figure S2a–d). Chemically, we can program the crosslinking density (*ϕ*_c_) at the outer and inner regions of the curved fiber affected by *R* and *κ*. Mechanically, the mode of locomotion is determined by the assembly of the fibers and the body, while the detailed movement is controlled by how mesogens are mechanically aligned. These sets of encoding dictate both thermodynamic and kinetic behaviors of the fiber deformation upon heating and recovery upon cooling.

LCE fibers are attached to a body as appendages and raise CoM. First, we emulate both the shape and functions of an octopus by attaching the fibers with a vertical rotational symmetry axis underneath the robot's body (Figure 1d,e). Next, we mimic the rolling morphology and motion of the golden wheel spider (Figure 1f) [2]. To prevent the robot from toppling, we adopt the spider's mirror symmetry design, where the locomotion can be customized in terms of speed, direction, and its dynamic variation over time; this rolling robot is hereafter referred to as ‘rollbot’ (Figure 1g). The chemomechanical encoding, in conjunction with the number of appendages (Figure S2e), allows for customization of the robot's locomotion. To systematically analyze the influence of each deformation mode, we decouple the effects of stretching imposed in Stage II alone.

### 2.2 Mechanical Properties of LCE Fibers

After the first crosslinking, *R*1.25 exhibits greater extension than *R*1.1 because *R*1.25 has a lower *ϕ*_c_ and a higher fraction of dangling chains resulting from the increased off‐stoichiometry. Thus, *R*1.25 possesses greater chain mobility for rearrangement during the stretching process, resulting in a larger achievable elongation before breakage. Stretchability also depends on the fiber's diameter. When fabricated using tubular molds with inner diameters of 3.17 mm (referred to as LD, for large diameter) and 1.58 mm (SD for small diameter), *R*1.1 fibers extend to 310% and 450%, respectively, while *R*1.25 fibers extend to 460% and 530%, respectively (Figure S3). The diameters are chosen to ensure the fibers have adequate stiffness to support the robot's entire body, while remaining thin enough to maintain a relatively fast deformation rate due to heat‐conduction constraints. The thinner fibers exhibit greater extension than their thicker counterparts, which is ascribed to dangling chains that are prone to migrate to the surface due to their lower conformational entropy [58]. The effect of dangling chains is amplified in SD fibers because of the larger surface‐area‐to‐volume ratio. These dangling chains help relax internal stress by dissipating more energy, thereby creating an environment that facilitates chain rearrangements [59].

UV crosslinking locks the structure, in which a higher fraction of dangling chains provides additional sites for crosslinking. Therefore, in the UV‐crosslinked, stretched‐only fibers, *R*1.25 has a greater Young's modulus (*E*, 44.7 MPa) than that of *R*1.1 (26.1 MPa), as measured by tensile tests (Figure S4a). The higher *ϕ*_c_ after the two‐step process in *R*1.25 enables the fiber to exhibit a larger force of contraction (Figure S4b), and a higher *T*_NI_, 95°C vs. 90°C for *R*1.1 (Figure S4c). Additionally, thickness matters; a thicker fiber generates a greater contraction force due to an increased number of mesogens.

### 2.3 Deformation and Recovery of the Twisted LCE Fibers

As the final form of LCE fibers incorporates tensile, torsional, and flexural components, we decompose each deformational contribution to analyze the influence of key factors: fiber diameter, twist density, *R*, and *κ*. The extension and shrinkage of fibers in the axial direction are not the primary focus of this study, as torsional and flexural deformation and recovery drive the robots’ movement. The fibers are stretched into a maximally strained, kinetically trapped state, wherein they store the elastic energy that drives them back to their original, random configurations. This step is crucial to ensure uniform twists, which would otherwise be unachievable due to partial necking. Here, we focus solely on the effects of twisting and bending, with the fibers pre‐strained.

**FIGURE 2:**
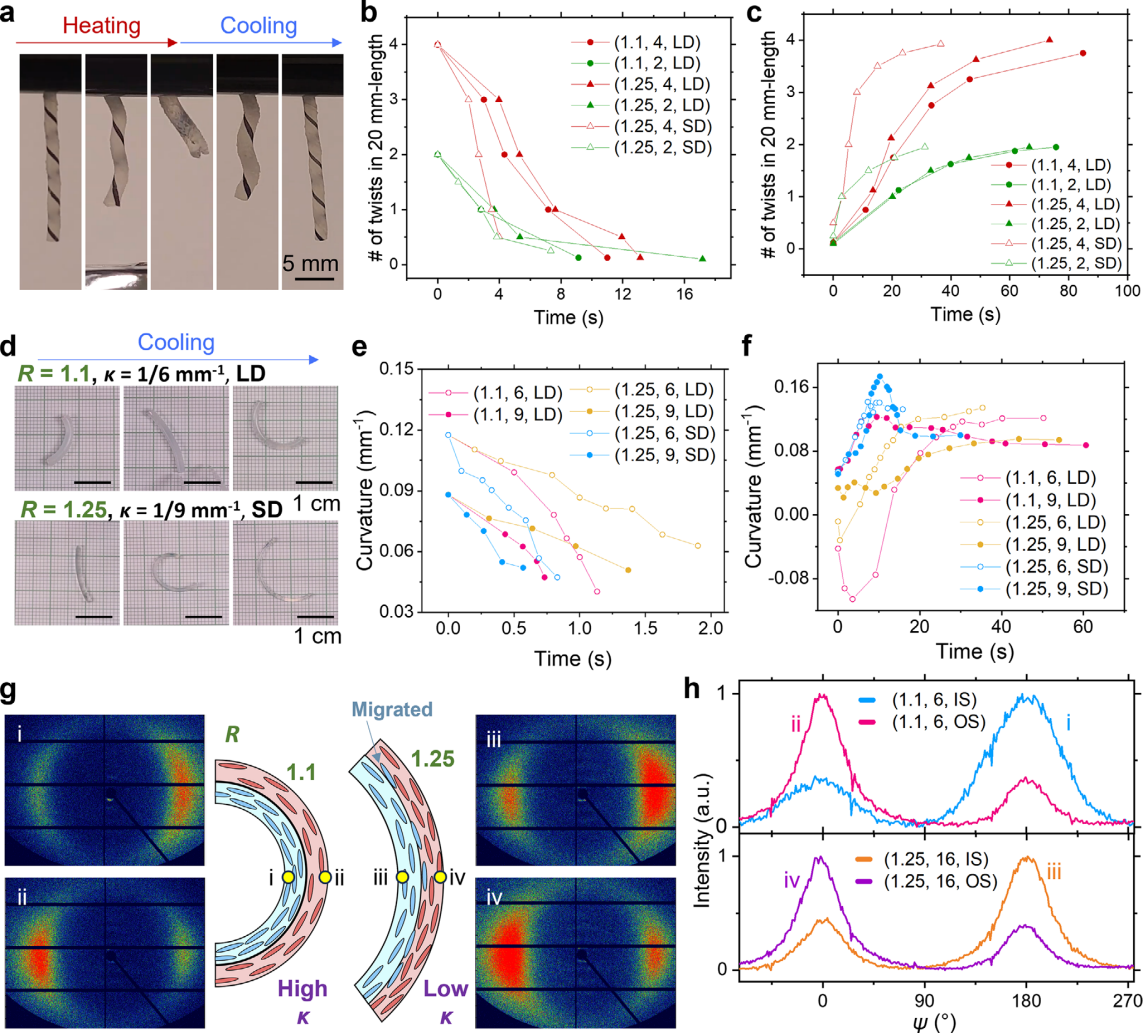
Torsional and flexural deformation and recovery by temperature. (a) Photos of a twisted LCE fiber upon heating to 220°C using a heat gun and cooling to room temperature. The black line indicates the number of twists. (b,c) Time‐dependent number of twists of LCE fibers upon heating (b) and cooling (c). The first two values are the *R* value and the initial number of twists in a 20‐mm‐long fiber. The terms LD and SD refer to large and small diameters, respectively. (d) Photos showing the recovery of a bent LCE fiber heated by a heat gun. (e,f) Time‐dependent curvature change of the LCE fibers upon heating in a 140°C silicone oil bath (e) and cooling (f). (g) Illustrations of the distribution of the mesogens in the bent LCE fibers (the center panel) and 2D XRD patterns of the fiber taken at positions (i) to (iv) (the left and right panels). High *κ*: 1/6 mm^−1^, and low *κ*: 1/16 mm^−1^. (h) The azimuthal scan of the XRD patterns corresponding to those in (g).

We examine the speed of deformation and recovery of the twisted fibers upon heating and cooling, varying the fiber diameter, twist density, and *R*; the fibers are heated using a heat gun along the longitudinal direction to prevent undesired oscillation and cooled at room temperature (Figure 2A). By counting the number of twists in a 20 mm‐long fiber, the untwisting and twisting time windows are observed (Figure 2b,c, Figures S5–S7 and Movie S2). Thinner fibers deform and recover more quickly, attributed to their faster heat absorption and dissipation. In addition, a lower *R* leads to faster deformation due to a lower *ϕ*_c_, but a higher *R* enables faster recovery due to the enhanced shape restorability; the surplus acrylate groups from the off‐stoichiometric composition form a dense secondary network upon photo‐crosslinking, which acts as a structural memory frame that promotes rapid elastic recovery to the original geometry. SD *R*1.1 is excluded from this study as its insufficient memory effect and significant energy dissipation—stemming from a low crosslinking density and surface dangling chains—prevent complete shape recovery during cooling (Figure S8) [60].

While insufficient photo‐crosslinking density leads to a noticeable initial recovery loss at 220°C, the fibers exhibit stable and repeatable performance over extended cycles. To validate the long‐term stability, we conduct a deformation‐recovery cyclic test for the torsional mode at 220°C on *R*1.1, which is expected to have the lowest performance, as it represents a worst‐case scenario for reversibility due to a low photo‐crosslinking density (Figure S9). When a straight 20 mm‐long fiber with 4 twists (twist angle, 1440°) is heated, the average twist angle from the first five cycles decreases to 1402°. After 100 cycles, the average twist angle from the final five cycles, 1383°, shows a minimal decrease. This indicates that the deformation behavior stabilizes significantly after the initial cycles, ensuring reliable and repeatable performance of the soft robots.

### 2.4 Deformation and Recovery of the Bent LCE Fibers

Next, LCE fibers that are bent but not twisted are examined (Figure 2d and Movie S3). When heated directionally in air with a heat gun, the bent fibers show a similar trend to the twisted ones, that is, the deformation speed is faster for lower *R* and smaller fiber diameter (Figure 2e). Given that a larger twist number results in faster untwisting, it is expected that a bent fiber with a larger *κ* would be straightened more quickly than one with a smaller *κ* upon heating. Instead, the fibers exhibit unexpected behaviors, including negligible change in *κ*, a temporary increase in *κ*, or further bending into the opposite curvature, depending on *R*, *κ*, and fiber diameter (Figure 2e,f). Since the fibers are not porous (Figure S10) [61], the bending behavior is likely governed by differences in the molecular network within the fiber. To better track the deformation kinetics, we place the LCE fiber in silicone oil at 140°C, which provides an isotropic heating environment and minimizes the effects of convective airflow from a heat gun. Overall, we observe two distinct anomalous behaviors in the bent fibers.

First, the LD *R*1.1 with a high *κ* of 1/6 mm^−1^ is straightened, then deformed to the opposite *κ* (Movie S4). This behavior is confirmed by observing the fiber's deformation and recovery both in hot oil (Figures S11 and S12a). Second, for SD *R*1.25 with a small *κ*, upon heating in oil, the curvatures of the fibers with initial *κ* = 1/9 mm^−1^ and 1/16 mm^−1^ unexpectedly increase (Figures S11c, S12b, and Movie S5). The phenomenon is obscured in the heat gun experiments, where a non‐uniform temperature gradient is introduced—the hot air is focused toward the outer (convex) region, mimicking the robot appendages’ initial ground contact (Figure 1e,g). Nevertheless, once the directional heat source is removed, the fiber exhibits its inherent response, with *κ* temporarily exceeding the initial value upon cooling (Figure S13). To elucidate these phenomena, we compare the strain magnitudes at each step of the fiber fabrication (Figure S14). When the demolded fiber (A) is stretched (A’), the strain *ε*_A’_ increases from the initial reference state (*ε*_A_ = 0). In the framework of classical rubber elasticity, the bending of an elastomer specimen induces a non‐uniform stress across its cross‐section. Specifically, the outer arc experiences significant tensile stress (Figure 2g), where the individual polymer chains undergo greater extension than those in the inner arc. Consequently, the strain at the outer arc (*ε*_B_) is larger than that at the inner arc (*ε*_C_). This deformation gradient, characterized by the disparity in network stretching, acts as a driving force that facilitates the migration of mobile dangling chains from regions of compression toward the highly strained outer arc [62]. As the fiber extends initially, *ε*_B_ and *ε*_C_ are both larger than *ε*_A_. However, during the bending process, the outer side is extended (*ε*_A’_ < *ε*_B_), while the inner side is contracted (*ε*_A’_ > *ε*_C_).

As an *R*1.25 contains more dangling chains than an *R*1.1, chain migration from the inner to the outer half‐arc is more extensive in *R*1.25 (C to B in Figure S14). Conversely, an *R*1.1 has a higher *ϕ*_c_ after the first crosslinking, limiting the chain migration. Consequently, the difference in the final alignment state between the inner and outer curved sides of a bent fiber must be smaller in *R*1.25, because migration reduces the extensive strain at the outer half‐arc. The evidence of migration is supported in the reduced XRD intensity contrast between the inner and outer half‐arcs in these anomalous fibers compared to that in *R*1.1 (Figure 2g). The two distinct diffraction spots from each edge are oriented perpendicular to the molecular alignment and exhibit different intensities, reflecting the increased sample volume through the thicker region of the fiber. When the spots are compared in terms of their respective intensities at opposite edges of the same fiber, *R*1.25 shows a much lower contrast than *R*1.1. From the azimuthal scans of the XRD patterns (Figure 2h), we calculate the order parameters (*S*), which quantify the degree of molecular alignment in the bent fiber, in both large and small peaks measured at the curved sides:

(1) $$S=\frac{3\langle \cos^{2}\varphi \rangle -1}{2}\;$$

(2) $$\langle \cos^{2}\varphi \rangle =\frac{\int_{-\frac{\pi }{2}}^{\frac{\pi }{2}}I\left(\varphi \right)\cos^{2}\varphi \left|\sin\varphi \right|d\varphi }{\int_{-\frac{\pi }{2}}^{\frac{\pi }{2}}I\left(\varphi \right)\left|\sin\varphi \right|d\varphi }\;$$

where *φ* denotes the azimuthal angle and *I*(φ) represents the XRD intensity as a function of φ. The calculated *S* values are summarized in Table 1. The ratios of the outer‐side *S* (*S*_OS_) to inner‐side *S* (*S*_IS_), *S*_OS_/*S*_IS_, for the *R*1.1 half arc are 1.136 and 1.131, respectively, while those for the *R*1.25 half arc are 1.022 and 1.006, respectively.

**TABLE 1:** Order parameters at the inner and outer sides of the bent LCE fibers and their ratios.

		*S*_IS_ (Inner side)	*S*_OS_ (Outer side)	Ratio (*S*_OS_/*S*_IS_)
***R*1.1** ***κ* = 1/6 mm^−1^**	**Small peaks**	0.509	0.578	1.136
	**Large peaks**	0.467	0.528	1.131
***R*1.25** ***κ* = 1/16 mm^−1^**	**Small peaks**	0.584	0.597	1.022
	**Large peaks**	0.531	0.534	1.006

The ratios imply that the outer‐arc edge of an LD *R*1.1 with a large *κ* (1/6 mm^−1^) has significantly better alignment than the inner‐arc edge. We hypothesize that, with limited molecular migration in *R*1.1, deformation gradient forces a significant extension of the outer half‐arc relative to the inner half‐arc, leading to a reduction in the degree of alignment from the outer to the inner arc (Figure S15). Correspondingly, the diffusion dynamics of the reactive moieties are suppressed, thus increasing the average intermolecular distances between mesogens along the alignment director [63]. Such diffusion‐limited kinetics hinder effective chain coupling during photo‐crosslinking, resulting in a lower *ϕ*_c_ in the outer arc. The *ϕ*_c_ contrast between the inner and outer sides of the curved fiber is further validated via atomic force microscopy (AFM) indentation tests on the cross‐section of the fibers (Figure S16). The inner arc of *R*1.1 is found to be stiffer than the outer arc, whereas *R*1.25 exhibits comparable moduli between the two sides. Consequently, the insufficiently fixed stretched state in the outer arc, characterized by reduced elastic confinement, leads to a dominant entropic recoil upon heating, ultimately reversing the curvature.

Accordingly, an SD *R*1.25 with a small *κ* (1/16 mm^−1^) would have smaller contrast of the alignment in the inner and outer arcs, because a larger fraction of dangling chains migrate toward the tensile outer arc. Furthermore, both a smaller diameter and a lower *κ* contribute to a smaller difference. It has been suggested that [62] while the polymer network of the outer arc expands under tension, the influx of mobile chains effectively fills the dilated interstices of the network, leading to a counterintuitive reduction in local volume through localized densification. Here, we propose that this high‐density distribution of dangling chains, once locked by photopolymerization, provides a significantly greater potential for thermal expansion upon heating [58]; the resulting disparity in free‐volume increase between the inner and outer arcs thus drives an unconventional increase in curvature. Finally, the reliable and reversible response of the bent fibers is also confirmed by a flexural deformation‐recovery cycle test with *R*1.1 (Figure S17). Although the initial recovery loss remains at 220°C, the fibers exhibit stable and repeatable performance over extended cycles. For a bent fiber with an initial curvature of 0.0975 mm^−^^1^, a slight relaxation occurs during the initial cycles at 220°C, reaching 0.0925 mm^−^^1^. However, after 100 cycles, the curvature stabilizes at 0.0866 mm^−^^1^. This result confirms that the flexural components, like the twisted ones, achieve the mechanical reliability necessary for consistent robotic actuation after a brief initial run‐in period.

### 2.5 Octopus‐Like Movement of LCE Robots

**FIGURE 3:**
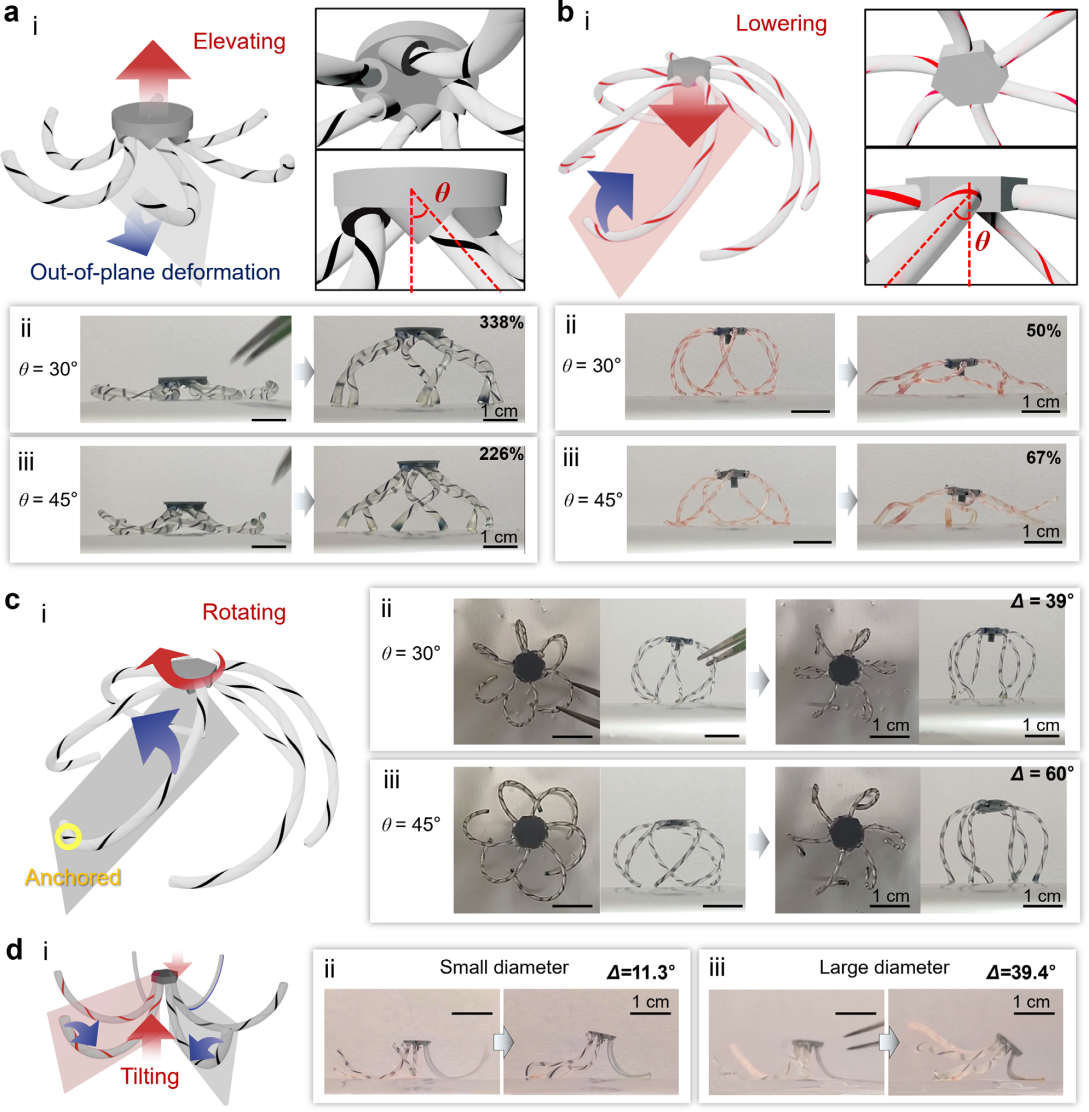
Locomotion of an octopus‐inspired soft robot. (a) Illustrations of a rising robot design (i) and its experimental photos with different *θ’*s (ii and iii). (i) The close‐up design of the 3D‐printed body and how fibers are attached to the body. The angle between the protruded appendage holder and the flat disk is defined as *θ*. Large‐diameter (LD) fibers of (*R*, 1/*κ* (mm^−1^), h # of twists per half loop) = (1.1, 9, 4) are used. (b) Illustrations of a lowering robot design. The angle between the flat side of the polygonal disk and the projection of the bent fiber onto the surface is defined as *θ*. Small‐diameter (SD) fibers of (1.25, 9, 4) are used. (c) Illustration of a rotating robot design (i) and its experimental photos with different *θ’*s (ii and iii). The design is the same as (b), but the handedness of the twist is the opposite. In each set, the left and right photos show the top‐view and side‐view of the robot, respectively. SD fibers of (1.25, 9, 4) are used. (d) Same set as (a) for a tilting robot design. All the fibers in (ii) have the same SDs with (1.25, 9, 4), while two of them are not twisted. The twisted and bent fibers in (iii) have LDs while other conditions are the same as (ii). All the hot surfaces are heated to 180°C.

Based on the understanding of thermodynamics and kinetics governing the deformation and recovery of the twisted and bent fibers, we design robots with individually addressable transformation modes to achieve specific functions. Although various locomotion modalities, including walking, swimming, crawling, climbing, and rolling, have been demonstrated by stretching, twisting, or bending fibers individually, they have yet to fully reproduce the complex, integrated movements in the arms of an octopus [57, 64, 65, 66]. Here, we emulate the movement of an octopus at two levels: the local deformation of LCE fibers (stretching, twisting, and bending) and the global body maneuvers (elevating, lowering, rotating, and tilting). The LCE fibers are encoded by mechanical alignment with twist and curvature. To achieve a rising motion, *R*1.1 fibers, which are twisted clockwise at a density of four twists per half‐loop, followed by bending, are secured to a central rigid‐body disk via other obliquely protruding disks, which are pre‐tilted counterclockwise beneath the center disk from the top view (Figure 3a(i)). The central disk serves as a critical mechanical junction that prescribes the boundary conditions for each appendage, which translates local fiber deformations into a synchronized structural response. Building on the out‐of‐plane deformation mechanism illustrated in Figure 1b and the material‐dependent behaviors characterized in Figure 2, the spatial organization of the appendages plays a key role in force integration. To ensure the untwisting fibers make effective ground contact and elevate the body without the need for a 90° rotation, small protruding disks—sized to match the LCE fibers—are printed with an attachment angle *θ* between the axes of the central disk and the fiber‐holding disk. By anchoring the fibers in a rotationally symmetric arrangement around the central disk, the individual twisting and unbending moments are coupled into a unified upward vector. This collective force generation is highly dependent on the alignment of the appendages along the rotational symmetry axis; such a configuration ensures that the localized stresses of each fiber reinforce one another rather than canceling out, thereby maximizing the mechanical advantage required for the body to rise (Movie S6).

To verify that the macroscopic rising motion is a consistent, inherently programmable response, we systematically analyzed the structural parameters that govern this vertical displacement. Specifically, *θ* is adjusted to control the degree of body rise. When *θ* = 30° and 45°, the robot ascends 338% and 226%, respectively, of its initial height, following the relationship *l*_c _cos*θ*, where *l*_c_ is the radius of curvature (Figure S18a–d). The fiber's intrinsic curvature is another factor that affects the extent of rise; lower‐*κ* fibers with other parameters fixed exhibit greater rise (Figure S18e,f). This behavior is attributed to the coupling and competition between deformation modes. Because the fiber ends are anchored to the substrate, the programmed unbending deformation is mechanically constrained, which dramatically increases the effective elastic resistance of the fibers [67, 68, 69] against raising the body, as seen from the higher‐*κ R*1.1 fibers. The ground‐contact angle of the *R*1.1 robot appendages with *κ* = 1/9 mm^−1^ (Figure S18d) is smaller than that of the *R*1.1 robot appendages with *κ* = 1/16 mm^−1^ (Figure S18f), indicating significantly less twisting.

We program different motions through the fiber positions. We attach the fiber's end to the side of a disk, so that the projection of the arc‐shaped fiber onto the disk's side face forms a straight line tilted by an angle *θ* from the vertical axis (Figure 3b). In this configuration, the twist handedness of the LCE fibers dictates the specific mode of postural change (lowering or rotating motion), despite the robot maintaining an identical morphology. With a counterclockwise twist, the anchoring point of each fiber shifts outward due to untwisting, as seen in the top view, thereby causing the body to be lowered (Figure S19a–d and Movie S7). The *θ* facilitates maintaining the untwisting fibers in close proximity to the hot surface, which promotes further deformation and subsequent lowering of the body. However, a larger *θ* results in a reduced initial height, thereby limiting the total extent of lowering motion. The robots with *θ* = 30° and 45° descend to 50% and 67% of their initial height, respectively, consistent with the geometric relationship 2*l*_c_sin*θ*. This is further confirmed by the fact that lower‐*κ* fibers, which are longer, achieve a greater descent (Figure S19e,f). With all conditions held constant but with the opposite twist handedness, the robot accomplishes a rotating motion. This is because the initially ground‐touching points become the fixed anchoring points, and the untwisting motion rotates the body. Larger *θ* causes greater rotation: 39° and 60° for *θ* = 30° and 45°, respectively (Figure 3c and Movie S8). The predictability of these postural changes further underscores the robust nature of our platform, as specific motion modes are deterministically programmed by the fiber configuration.

Unlike the soft robots reported in the literature [42, 54], which use synchronized deformation to generate a collective force, an octopus independently manipulates its arms to achieve versatile movements. As our robot uses independent appendages attached to a body, it can leverage different twist handedness programmed into each fiber to cause the body to deviate from its symmetry axis. We position two *R*1.25 fibers with a clockwise twist, two with a counter‐clockwise twist, and two with no twist in a clockwise sequence, all of which are extended and bent. Here, the fiber positions are kept with vertical rotational symmetry (Figure 3d). For the bent‐only fibers, we use SD *R*1.25 with *κ* = 1/9 mm^−1^ to exploit their anomalous behavior of further bending upon heating. Their further bending on the surface helps a tilting motion, combined with the other four fibers’ untwisting, which pushes the ground and raises one side of the body (Movie S9). As thicker fibers generate a larger actuation force (Figure S4b), the tilting angle of the twisted fibers increases from 11.3° (twisted SD fibers) to 39.4° (twisted LD fibers).

### 2.6 Golden Wheel Spider‐Like Movement of LCE Rollbots

**FIGURE 4:**
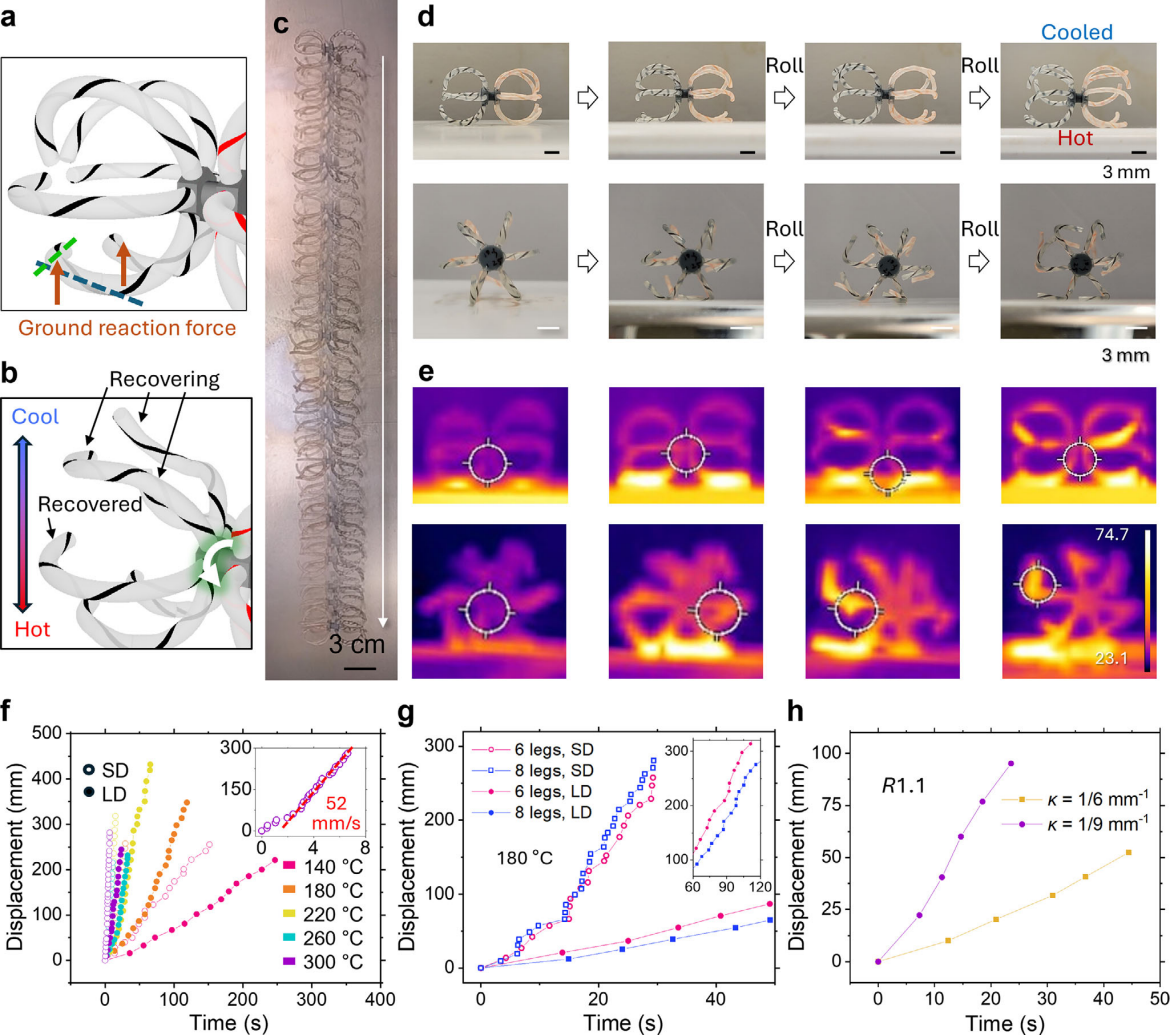
Rolling locomotion of the rollbot. (a,b) Illustrations of the robot's initial rolling step (a) and its sustained motion over several subsequent steps (b). (c) Overlaid images of straight‐line rolling of the same rollbot on a 100°C hot plate. (d),(e) Time‐lapse front and side views of a rolling rollbot shown using a digital camera (d) and a thermal camera (e). The fibers are encoded as (1.1, 4, 9, 6). (f) Displacement vs. time plot for (1.25, 4, 9, 6) rollbots on a hot surface at 140, 220, and 300°C. The inset shows the locomotion of an SD rollbot on a 300°C surface. (g),(h) Displacement vs. time plot for (1.25, 4, 9, 6) and (1.25, 4, 9, 8) rollbots (g) and for (1.1, 2, 6, 6) and (1.1, 2, 9, 6) rollbots (h) on a 180°C surface. The inset shows the extended time range for the LD rollbots.

Beyond octopus‐inspired posture‐changing maneuvers, we further extend the robot's capabilities by emulating the unique rolling locomotion of the golden wheel spider. The robot design proposed in Figure 1g, which we refer to as a rollbot, is designed to achieve autonomous rolling on a hot surface. The CoM of the rollbot is located at 0.30 times the body length (BL) along the axis parallel to the rotation axis. This value is significantly higher than that of most reported rolling LCE robots. For comparison, typical twisted LCE strips and coils exhibit much lower CoM‐to‐body‐length ratios of approximately 0.022 and 0.095, respectively. Two groups of twisted, bent fibers with opposite handedness are attached to each side of the cylindrical body. The untwisted fibers push against the ground, and the ground pushes back on the fibers. This elevated CoM intentionally creates a top‐heavy configuration that maximizes gravitational instability, effectively lowering the energy barrier required to convert the fibers' push into a continuous forward rolling moment. This leads the robot to rotate about its horizontal rotational symmetry axis and sustain the temperature‐gradient‐driven deformation cycle (Figure 4a). The deformed fibers are then lifted to the air, which is cooler than the surface, and begin to recover their original shape (Figure 4b). The recovered fibers are ready to deform again when they make contact with the hot surface after a period of rolling. This temperature gradient drives continuous rolling locomotion (Figure 4c and Movie S10), as confirmed by optical and thermal images (Figure 4d,e).

To elucidate the programmatic control and mechanical reliability of our platform, we conducted a comprehensive parametric study (Figure S20), revealing that the rollbot speed is strictly governed by design variables including *R*, twist density, curvature, and the number and diameter of fibers. Hereafter, each rollbot is specified by four parameters in parentheses as follows: (*R*, the number of twists in half‐loop, 1/*κ*, the number of appendages per side). A lower *R* and a larger twist density lead to a faster rollbot, due to lower *ϕ*_c_ after photo‐crosslinking and a higher degree of alignment, respectively (Figure S21a). Interestingly, the *R*1.25 rollbots exhibit a continuous increase in speed with temperature, from 140°C to 300°C (Figure 4f). Here, it is noted that *R*1.25 rollbots do not roll on a 100°C hotplate, whereas *R*1.1 rollbots do, albeit with a *T*_NI_ below 100°C. Since the LCE fibers are completely decoupled, the deformation of only four fibers in contact with the ground must generate sufficient force to actuate the entire body with 12 to 16 fibers. This configuration demands a much greater deformation force than a conventional LCE robot, thereby increasing the thermal threshold required to trigger a full rolling over.

At operating temperatures of 140°C, 220°C, and 300°C, the LD *R*1.25 (1.25, 4, 9, 6) rollbots reach speeds of 1.08, 7.79, and 11.40 mm/s, while the SD variants exhibit significantly higher speeds of 1.68, 27.32, and 51.88 mm/s. These speed values are measured after the rollbots complete one cycle, since they tend to accelerate in the second cycle as the fibers do not need to be fully recovered. Hereafter, all reported speeds correspond to the steady‐state values measured after the first full cycle. The fastest speed of 52 mm/s corresponds to 1.3 BL/s, normalized by the longer BL parallel to the rotation axis. In contrast, most reported LCE robots have a locomotion speed of less than 1 BL/s due to their low CoM profile and a small turning radius. Such behaviors are not observed in other twisted LCE strips because they untwist completely above 220°C [42]. Our rollbots overcome this limitation, as the deformed and raised fibers recover their initial shape at a distance 1/*κ* mm from the hot plate in the air, an environment that is significantly cooler, to enable the next rolling cycle. The Ashby plot analysis across the range of LCE‐based soft robots made from fibers, strips, and rods demonstrates that our rollbots achieve high relative speeds with a small cross‐sectional perimeter (Figure S22). As the operating temperatures of LCE robots vary across studies in the literature, depending on their *T*_NI_, we here compared the reported peak speed of each robot to provide a performance benchmark across their respective optimal operating regimes. The cross‐sectional perimeter was selected as a key axis for the Ashby plot; its reduction is particularly critical for rectangular geometries, as a smaller perimeter effectively minimizes the physical footprint at the surface interface. However, it makes it difficult to support high CoM structures. Reduced LCE material usage also compromises the number of mesogens that drive the movement. This performance trade‐off was overcome by adopting a bio‐inspired spider‐like architecture combined with the use of high‐*R* LCE fibers.

Fiber diameter leads to a starker difference in the rollbot speed; (1.25, 4, 9, 8) with SD fibers rolls at 13.7 mm/s, while that with LD fibers rolls at 3.6 mm/s. More importantly, the rollbots can take advantage of inertia, which allows them to continue rolling two or more steps at a time. The rolling inertia is enhanced by the combination of a high CoM and a minimal contact footprint. Specifically, the round cross‐section of the LCE fiber ensures that each successive engagement with the surface occurs via only two discrete point contacts rather than a broad planar interface. This minimized contact area effectively reduces ground effects, allowing the high CoM rollbot to drive the rolling momentum more efficiently. The multiple‐step rolling is confirmed in Figure 4g, where the group of dots exhibits a steeper slope between intermittent delays. As the SD fibers deform faster than LD fibers, their robots are more susceptible to inertia. Finally, the effect of fiber curvature on the rollbot's locomotion is influenced by the anomalous deformation and recovery behaviors of bent LCE fibers. Anchoring the ends of the twisted and bent fibers to the surface is crucial, as it enables the untwisting motion to exert a propulsive force against the hot surface. Here, the speed of unbending is critical because the fiber must remain bent to exert a large pushing force onto the surface without spinning. When the unbending is too fast, the fiber tends to spin in place and requires a larger degree of bending to complete a single rolling step. However, the anomalous behavior of the bent *R*1.1 with *κ* of 1/6 mm^−1^ makes the rollbot roll slower than one with *κ* = 1/9 mm^−1^. A similar trend is observed for *R*1.25 rollbots. However, their speeds are comparable, because the deformation speed of *R*1.25 with *κ* of 1/6 mm^−1^ is faster than that of fibers with smaller curvatures (Figure S21b).

**FIGURE 5:**
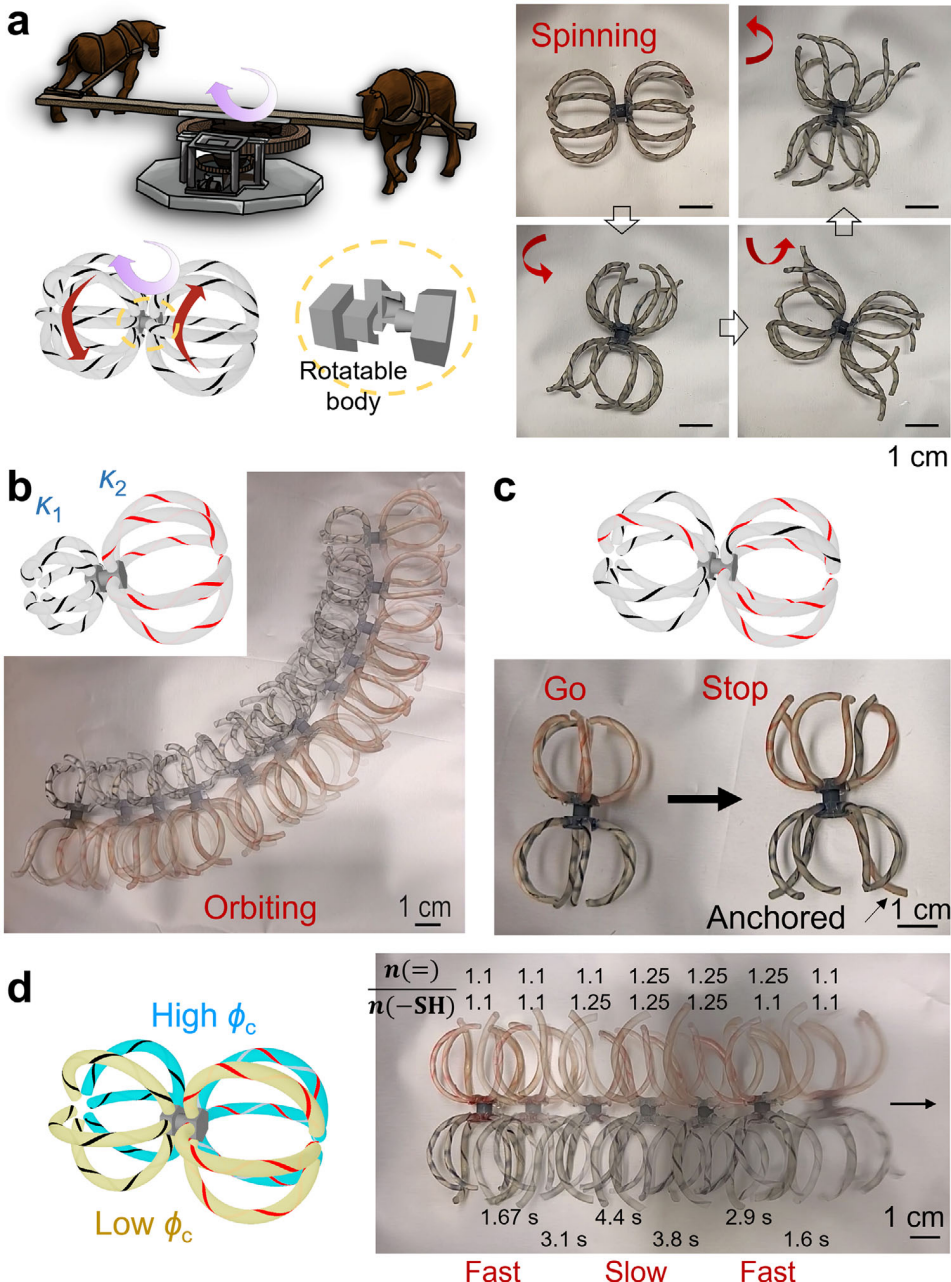
Rolling locomotion of rollbots. (a) Illustration of a horse mill, a spinning rollbot design (left), and the corresponding experimental photos (right). The rollbot is encoded as (1.25, 4, 16, 8). (b) Orbiting rollbot. One side is encoded as (1.25, 3, 6, 6), while the other side is encoded as (1.25, 3, 9, 6). (c) Rollbot with a go‐stop motion. A (1.25, 4, 9, 6) rollbot has a single fiber on each side with opposite handedness and the same angle. (d) Rollbot with a dynamically changing speed. Three sets of fibers are (1.25, 4, 9, 6) in a row, and the other three sets are (1.1, 4, 9, 6).

The rollbot's locomotion can be customized not only in speed, but also in direction and the dynamic change of its motion by altering the body design or the fiber assembly. For example, a spinning motion is achieved by attaching fibers with the same‐handed twists onto a 3D‐printed body with two separate, rotatable parts (Figure 5a and Movie S11), much like a horse mill, which can be used for energy storage. When the *κ*s of fibers on the two sides of the body are set differently, the robots show an orbiting motion (Figure 5b). The rollbot can also stop after a single rolling step if two fibers with opposite twist handedness are included in one step (Figure 5c). As the opposite‐twist fibers are anchored to the surface, the forces they generate counteract each other, which prevents the rollbot from proceeding. Finally, a rollbot demonstrates dynamically changing speeds by using two different *ϕ*_c_s in half of the fibers in a row on each side (Figure 5d). As *R*1.1 deforms faster than *R*1.25, the rollbot's speed changes dynamically: it rolls faster when *R*1.1s are working to propel it forward and slower during the subsequent three steps when *R*1.25s are engaged.

### 2.7 Adaptability of the Rollbots in Various Environments

**FIGURE 6:**
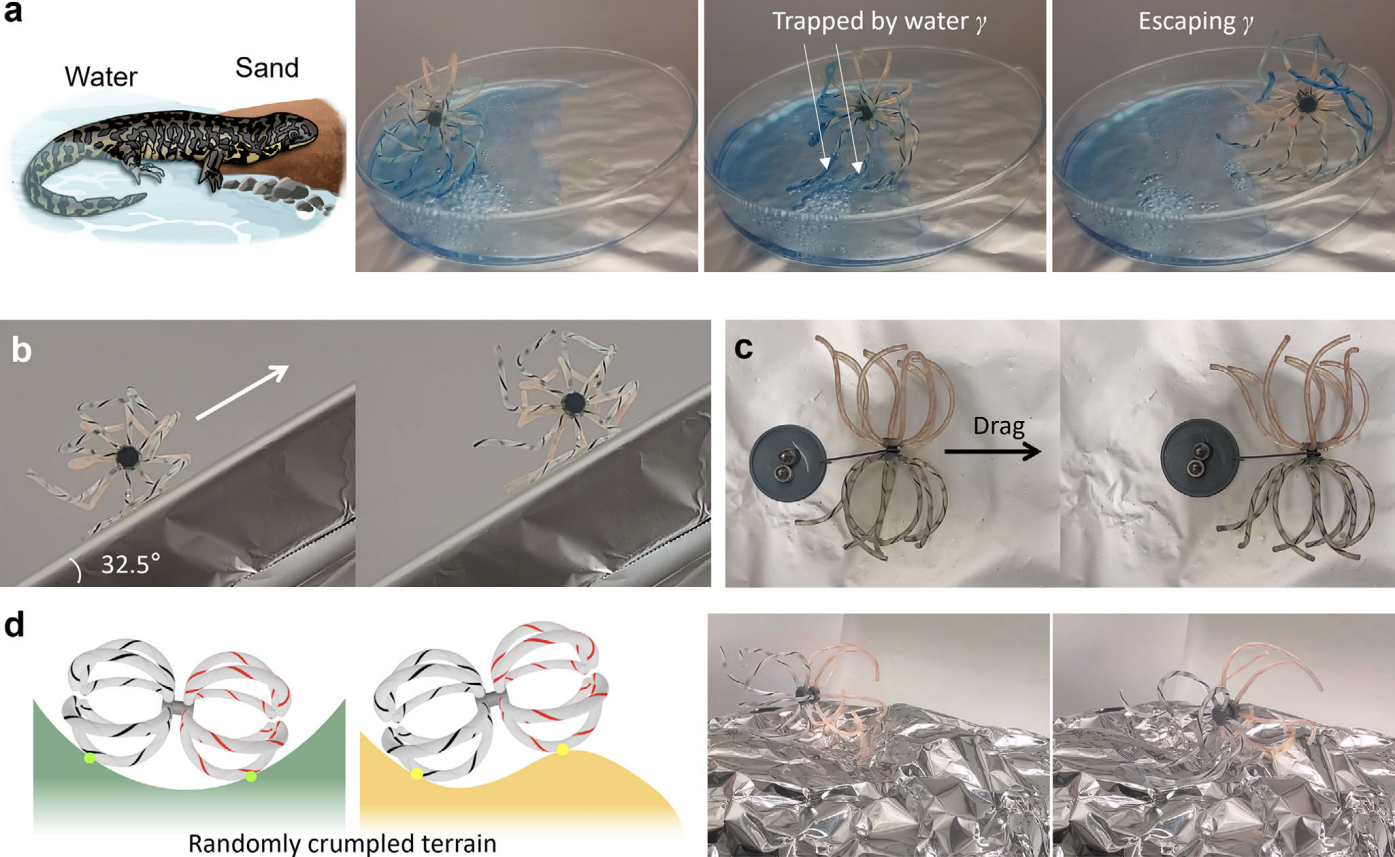
Adaptive locomotion of rollbots across diverse terrain and environments. (a) Illustration of a lizard for analogy of the transition from aquatic to terrestrial environments (leftmost panel) and time‐lapse photos (right panels) of a (1.25, 4, 16, 8) rollbot moving from 95°C water to land, demonstrating its escape from surface tension forces. (b,c) Time‐lapse photos of the same robot's climbing on a 32.5° ramp (b) and its dragging locomotion with a 4.8 g cargo (c). The surface temperature is 180°C. (d) Illustrations of the rollbot's point contacts with randomly crumpled terrain (left) and time‐lapse photos of the rollbot crossing the terrain at 180°C (right).

Our rollbots feature three key design principles: 1) a high CoM, 2) firm anchoring of the appendages to the surface via out‐of‐plane deformation, and 3) minimal point contacts with any terrain due to their semicircular‐arc geometry. They leverage these features to adapt to and move on various environments that are impassable for other types of LCE robots. First, a high CoM allows a rollbot to move in heated shallow water, as the deformed fibers can recover their shape once lifted above the water (Figure S23a). Previous LCE robots have been shown to travel on the water's surface [39] or underwater [18, 28]. However, surface tension could also restrict the robots from moving away from the water‐air interface. This issue has been addressed in other types of soft robots through combustion [70] or magnetic‐field control of individual body segments [71]. Our rollbot can escape water by a strong anchoring force provided by the LCE fibers against the ground, where one end of the fiber gradually deforms and conforms to the hot surface, similar to the amphibious reptiles that push off a surface (Figure 6a). The other end, in turn, rotates the body via a torsional force. Once the anchored fibers push the rollbot upward, the high CoM and the other forward‐tilted appendages help the rollbot overcome surface tension (Movie S12). With the same mechanism, the (1.1, 4, 16, 8) rollbot can climb stairs with a step height of 6 mm (Figure S23b).

Further, our (1.25, 4, 16, 8) rollbot successfully climbs a 32.5° ramp (Figure 6b and Movie S13). In contrast, other LCE robots typically roll on ramps with slopes of 10°–15° in the absence of effective anchoring [18, 42, 46], or a ratchet surface is needed to assist the anchoring of the LCE strip [72]. The individual appendage fails to continuously roll up the ramp, highlighting the necessity of the assembled rollbot body (Figure S24) to leverage a strong anchoring mechanism and the cooperative pushing force from multiple fibers. When linking the body to a cart loaded with weights (Figure 6c and Movie S14), the (1.25, 4, 16, 8) rollbot successfully drags a 4.8 g cargo—2.2 times its own weight—on a 180°C surface, with the dragging force reaching 13.3 mN, considering the friction factor of 0.28. In comparison, the (1.1, 4, 16, 8) rollbot drags a lighter cargo of 3.8 g (drag force, 10.53 mN), which can be explained by the greater stiffness of *R*1.25 and the resulting manifestation of higher force (Figure S4a,b). In the meantime, the (1.25, 4, 9, 8) rollbot drags a 2.1 g cargo (drag force, 5.82 mN), which indicates that a smaller curvature (i.e., a larger radius of curvature) is advantageous for exerting force, due to a longer length of mesogens participating in the actuation and a higher CoM.

Lastly, the rollbots can navigate terrain featuring randomly sized grooves and protrusions via minimal point contact between the surface and semicircular‐arc‐shaped fibers. This terrain is created by crumpling an aluminum foil and placing it on a 220°C hot plate (Figure 6, Figure S25, and Movie S15). While maintaining a total of four contact spots, each rolling step forms two new point contacts, and these fibers conformally deform to match the ground topography. Therefore, a continuous untwisting force propels the rollbot to achieve rolling on the terrain. The rollbot can climb ascending slopes or negotiate irregularities within the random terrain, as shown in its performance on ramps and stairs. It can also roll quickly down descending slopes, much like the golden wheel spider that utilizes gravity for faster locomotion. Even more, when the appendages are initially embedded in the sand, their simultaneous torsional and flexural deformations create a digging effect, enabling the robot to excavate objects such as a buried U.S. quarter (Figure S26).

## 3 Conclusion

In this work, we demonstrate highly maneuverable, adaptable, and customizable LCE‐based leggy robots inspired by an octopus and a golden wheel spider. The high adaptability is rooted in the LCE's replication of an animal‐like morphology, specifically the free‐ended appendages and high CoM. Unlike the monolithic LCE robots [42, 43, 49, 54], the appendages in our robots have one free end, allowing the body to move with maximum excursion in any direction, depending on the robot's design. The deformation is primarily a combination of torsional and flexural motions, which generate a synergistic force that propels the body in a direction not shared by any individual fiber. This is enabled by the rotational symmetry of the fiber positions, which allows the fibers’ propulsion vectors to sum and generate movement in a target direction. Finally, the fiber's bent geometry plays a crucial role in the robots’ locomotion and adaptability. It promotes strong anchoring to the surface, as the untwisting motion fixes the partially unbent fiber onto the substrate. Furthermore, bent fibers allow for point contact between the robots and the surface on any random terrain. A reduced contact area enhances mobility, a benefit further amplified by a high CoM. Interestingly, the rollbots take advantage of their high CoM and the resulting inertia to achieve higher speed. The aforementioned features enable the rollbot to seamlessly traverse diverse environments, including shallow water, a 32.5° ramp, stairs, and terrain with random crumpling.

Next, the intrinsic customizable nature stems from the diversity in the mechanical alignment of mesogens, their chemical composition, and the assembly of LCE fibers. More specifically, *R*, twist density, *κ*, number, diameter of LCE fibers, and the surface temperature affect the robots’ movement. We analyzed the effects of each factor on the deformation and recovery of LCE fibers, which help tailor the extent and speed of the robot's locomotion. All the extents of lifting, lowering, rotating, and tilting of octopus‐inspired robots were demonstrated to vary with different parameters in the robots. Likewise, the rolling speed of golden wheel spider‐inspired robots can range from 0.88 to 52 mm/s depending on the parameters. Their movement direction and speed variations can also be programmed for customization. Most importantly, the combined effects of dangling chains and curvature on deformation and recovery with temperature were examined using both XRD and the observed mechanical behavior of the LCE fibers. Two newly discovered anomalous behaviors defy straightforward expectations on deformation, providing a deeper understanding of the robot's movement and its dependence on each parameter. We expect these findings to enable the future design of the winding behavior of tendril‐shaped coiled fibers and the curvature inversion of smart valves, respectively, once these LCE fibers are fabricated using mechanical alignment and bending.

Most LCE research has focused on mimicking the muscle's working mechanism at the microscale [73, 74] and on achieving various forms of actuation with optimal LCE geometries, but not on mimicking the overall shapes of living creatures. The basic actuation modes of a fiber include contraction/elongation [19], twisting/untwisting [36], and oscillation [38], but the fibers are typically tethered to a payload to control and stabilize the actuation. Thus, control of recovery receives limited attention. In our study, we observed that the deformation and recovery kinetics of the bent LCE fibers involve a delayed stabilization period characterized by temporary fluctuations, particularly during the recovery phase. These complexities likely arise from the interplay between molecular migration and disparities in crosslinking density, resulting in longer response times than in simpler LCE systems. In this work, LCE fibers have only one end fixed, achieving a higher degree of freedom in locomotion that mimics the movements of an octopus and a golden wheel spider. While our design can be further improved for reprogrammability, the limitations of current work also highlight a critical area for future soft robotics research: the precise control of transient kinetics to achieve multimodal and reversible locomotion. Optimizing material compositions, e.g., by incorporating side‐chain liquid crystal monomers to create nematic‐smectic micro‐phase separations [75], extending thiol spacer lengths [76], or introducing bulky pendant groups to reduce the order of molecular packing [65], can effectively lower the actuation temperature threshold to near‐ambient or physiological ranges (35°C–65°C). Such thermal‐responsive tuning will broaden the uses of LCE actuators for wearable smart textiles, biomedical applications, and minimally invasive surgical tools. Meanwhile, adopting a core–shell structure with a passive yet highly elastic core and a responsive LCE shell could enable an ultrafast, highly elastic response while reducing the initiation temperature [77]. Ultimately, this study demonstrates a new design paradigm in which the synergistic interplay between material properties and bio‐inspired morphology can be leveraged.

## 4 Experimental Section/Methods

### 4.1 Materials

1,4‐bis‐[4‐(6‐acryloyloxyhexyloxy)benzoyloxy]‐2‐methylbenzene (RM82, 98%) was purchased from Henan Wentao Chemical Product Co., Ltd. 2,2′‐(Ethylenedioxy)diethanethiol (EDDT, 95%), pentaerythritol tetrakis(3‐mercaptopropionate) (PETMP, >95%), 2‐hydroxy‐4′‐(2‐hydroxyethoxy)‐2‐methylpropiophenone (HHMP, 98%), and diphenylamine (DPA, ACS reagent grade, ≥99%) were purchased from Sigma–Aldrich. Polydimethylsiloxane (PDMS, Sylgard 184) was purchased from Dow Corning. Toluene (analytical reagent grade) and silicone oil (for melting point and boiling point apparatuses) were purchased from Fisher Chemical. Two types of silicone tubing (1/8″ ID X 3/16″ OD and 1/16″ ID X 1/8″ OD) were purchased from Metaland.

### 4.2 First Crosslinking of LCE Fibers

The one‐pot method and the mechanical alignment utilized in this work consist of three stages (Stage I, II, III). For Stage I, 2 g of RM82 and 0.7 g toluene were added into a vial, which was subsequently put in a 90°C convection oven until the mesogen was fully dissolved. After the dissolved mesogen was cooled down, we added 0.368 g EDDT, 0.164 g PETMP, and 12 mg HHMP for *R*1.1, and 0.325 g EDDT, 0.145 g PETMP, and 12 mg HHMP for *R*1.25. The vial was put into the oven again till all the chemicals were fully dissolved. When the vial cooled down to room temperature, 0.25 g of 2 wt.% DPA solution in toluene was added and thoroughly mixed. The solution was injected into silicone tubing with an inner diameter of 1/8″ for LD LCE fibers and that of 1/16″ for SD LCE fibers. We waited overnight for the Michael addition reaction to complete and for the toluene to evaporate at room temperature. The fibers were demolded from the tubing and placed in a 70°C oven overnight to evaporate the remaining toluene.

### 4.3 Second Crosslinking of LCE Fibers and Mechanical Alignment

For Stage II, a long straight line was drawn on the LCE fiber as a marker to track the twisting deformation. We used black for clockwise twisting, red for counter‐clockwise twisting, and blue for no twisting. The LCE fiber was stretched to the maximal elastic strain, a point at which it is kinetically trapped without spontaneous shrinkage at room temperature. However, stretching beyond this point reveals a secondary elastic region, and the fiber returns to the maximal strain upon release of the force. It was twisted and subsequently wound around cylinders with radii of 6 mm, 9 mm, and 16 mm to induce bending deformation. After being fixed on the cylinder using Scotch tape, it was photo‐crosslinked by UV for Stage III. The UV curing process was repeated after the cylinder was rotated to expose the shaded part of the fiber, and again after the fiber was cut into a semicircular arc.

### 4.4 Atomic Force Microscopy (AFM) Indentation Tests

AFM‐based mapping and indentation tests were conducted by Asylum MFP 3D AFM. The HQ: NSC15/Al BS probe manufactured by MikroMasch was selected. The spring constant of the probe cantilever was around 40 N/m. For sample preparation, the LCE fiber was cut into small pieces and attached to a silicon wafer using carbon tape. A typical AFM indentation was conducted at a loading rate of 400 nm/s.

### 4.5 Fabrication of the LCE Robots

The center body was 3D‐printed by a digital light processing method (Sonic Mini 8K S, Phrozen) using a rigid resin (Aqua‐Gray 8K, Phrozen). For a dumbbell‐like geometry of a rollbot body, two disks with vertically positioned male and female linkers were 3D‐printed and subsequently linked. The 3D‐printed body contained markers that indicate both the attachment points and the precise angle for each fiber. To achieve a targeted design, the LCE fibers were attached to the body with a photo‐crosslinkable bond (Photobond GB368, Delo) using a hand‐held UV light (365 nm, 735 mW/cm^2^, CS2020, Thorlabs).

### 4.6 Analysis of Fiber Deformation and Recovery

We used two heating methods: a heat gun and a hot oil bath. Deformation was induced by heating with a heat gun (ROMECH‐141) at a distance of 1 cm, with an air output volume of 66 gallon/min and a temperature of 220°C (a reading from the heat gun, which might not be equivalent to the actual temperature experienced by the LCE fiber). Upon removal of the heat gun, the cooling process began. For the heating and cooling process with oil, two oil baths were prepared at 140°C and room temperature, respectively. The fibers were put into hot oil and then room‐temperature oil subsequently.

### 4.7 X‐Ray Diffraction Experiments

LCE fiber alignment was characterized using the Dual‐source and Environmental X‐ray Scattering (DEXS) facility at the University of Pennsylvania. Samples were measured using a Xuess 2.0 from Xenocs with a GeniX3D S4 source (Cu K𝛼, *λ* = 1.54 Å) and a Pilatus3R 1 m detector. Measurements were taken in a transmission configuration with a sample‐to‐detector distance of ∼172 mm and an exposure time of 300 s, wherein the fibers were placed vertically relative to the 2D detector. To measure the left and right sides of an LCE fiber, the following steps were taken: (1) the diameter of the fiber was first measured with a scan (∼0.1 s exposure) across the axes of the sample stage, (2) based on the resulting intensity map of the sample stage, the center of the filament was identified and another scan was taken across the diameter of the fiber (50 points across the fiber diameter, ∼0.1 s exposure), and (3) based on the resulting intensity map of the LCE filament, the left and right sides of the filament were measured as ∼0.094 mm (large diameter) or ∼0.045 mm (small diameter) from the edges of the filament. The edges of the filament were defined as points on the map where intensity was maximal (i.e., the filament is no longer within the beam).

### 4.8 Characterization

LCE fibers were characterized using a scanning electron microscope at an acceleration voltage of 5 kV in secondary electron mode (7500F HR‐SEM, JEOL). The tensile test and the measurement of contraction forces of LCE fibers with diameters of 0.75 mm (SD) and 1.60 mm (LD) were conducted by a universal testing machine equipped with a 10 N load cell (Instron 5564, Instron Corporation). The fibers were tested at a constant strain rate of 5 mm/min. The nematic‐to‐isotropic transition temperatures were measured by differential scanning calorimetry (DSC, Q2000, TA Instruments). Samples weighing approximately 5 mg were sealed in aluminum pans and analyzed under a constant flow of nitrogen and helium (50.0 mL/min each) at a heating rate of 10°C/min. *T*_NI_ was observed at the second cycle. Infrared (IR) thermographs were recorded using a compact thermal imaging camera (FLIR C5, Teledyne FLIR).

## Author Contributions

J.B.K. and S.Y. conceived the ideas and designed the research. J.B.K. fabricated LCE fibers and robots, conducted their characterization, and demonstrated the locomotion of robots. J.B.K. and A.P.M. performed XRD characterization. Y.H. and Z.Z. devised LCE material systems. Y.H. devised a theoretical background of mechanics. J.B.K. and Z.Z. conducted a tensile test. K.‐Y.W. conducted an AFM indentation test. S.Y. supervised the project. J.B.K. and S.Y. wrote the manuscript. All authors participated in discussions and reviewed the manuscript.

## Funding

National Science Foundation (NSF)’s Materials Research Science and Engineering Center (MRSEC) at the University of Pennsylvania (#DMR‐2309043, to S.Y.). Future Eco Manufacturing Research Grant (FMRG, #CMMI‐2037097, to S.Y.). National Research Foundation of Korea (NRF) funded by the Ministry of Education (RS‐2025‐02654033, to J.B.K.).

## Conflicts of Interest

The authors declare no conflicts of interest.

## Supporting information




**Supporting File 1**: adma73794‐sup‐0001‐SuppMat.pdf.

**Supporting File 2**: adma73794‐sup‐0002‐MovieS1.mp4.

**Supporting File 3**: adma73794‐sup‐0003‐MovieS2.mp4.

**Supporting File 4**: adma73794‐sup‐0004‐MovieS3.mp4.

**Supporting File 5**: adma73794‐sup‐0005‐MovieS4.mp4.

**Supporting File 6**: adma73794‐sup‐0006‐MovieS5.mp4.

**Supporting File 7**: adma73794‐sup‐0007‐MovieS6.mp4.

**Supporting File 8**: adma73794‐sup‐0008‐MovieS7.mp4.

**Supporting File 9**: adma73794‐sup‐0009‐MovieS8.mp4.

**Supporting File 10**: adma73794‐sup‐0010‐MovieS9.mp4.

**Supporting File 11**: adma73794‐sup‐0011‐MovieS10.mp4.

**Supporting File 12**: adma73794‐sup‐0012‐MovieS11.mp4.

**Supporting File 13**: adma73794‐sup‐0013‐MovieS12.mp4.

**Supporting File 14**: adma73794‐sup‐0014‐MovieS13.mp4.

**Supporting File 15**: adma73794‐sup‐0015‐MovieS14.mp4.

**Supporting File 16**: adma73794‐sup‐0016‐MovieS15.mp4.

## Data Availability

The data that supports the findings of this study are available in the supplementary material of this article.
