# Supplementary material for: High Center‐of‐Mass, Multi‐Legged Soft Robots Powered by Geometrically Encoded Liquid Crystal Elastomer Arc Appendages
Source: Adv Mater. 2026 Jun 19;38(42):e73794. doi: 10.1002/adma.73794 (PMC13410795; doi:10.1002/adma.73794)
Supplement: Supplementary file 1 — Supporting File 1: adma73794‐sup‐0001‐SuppMat.pdf. [file ADMA-38-e73794-s009.pdf]

Supporting Information

High Center-of-Mass, Multi-Legged Soft Robots Powered by Geometrically Encoded Liquid Crystal Elastomer Arc Appendages

*Jong Bin Kim, Antonio Proctor Martinez, Yaoye Hong, Ziyun Zhang, Kun-Yu Wang, and Shu Yang**

*Corresponding Author: shuyang@seas.upenn.edu

This file includes:

Figures S1 to S26

Legends of Movies S1 to S15

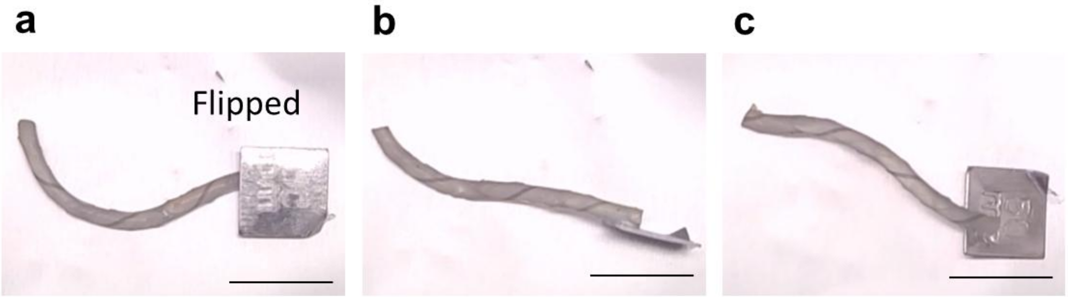

Figure S1. Subsequent deformation of the twisted and bent *R*1.25 fiber after Figure 1C. (a–c) Out-of-plane deformation with a steel plate attached to the end, making the plate flip twice with time.

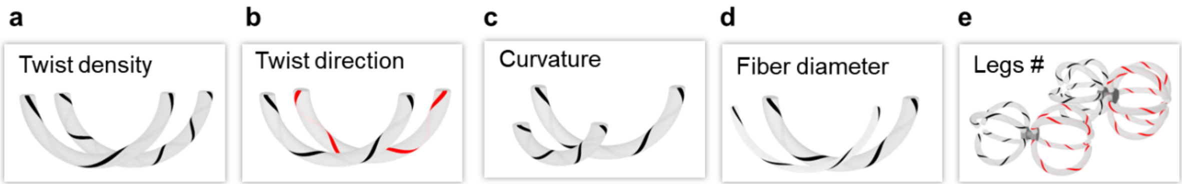

Figure S2. Variations that soft robots can have with LCE fibers. (a–e) Illustrations showing the variations in twist density (a), twist direction (b), curvature (c), fiber diameter (d), and the number of legs (e).

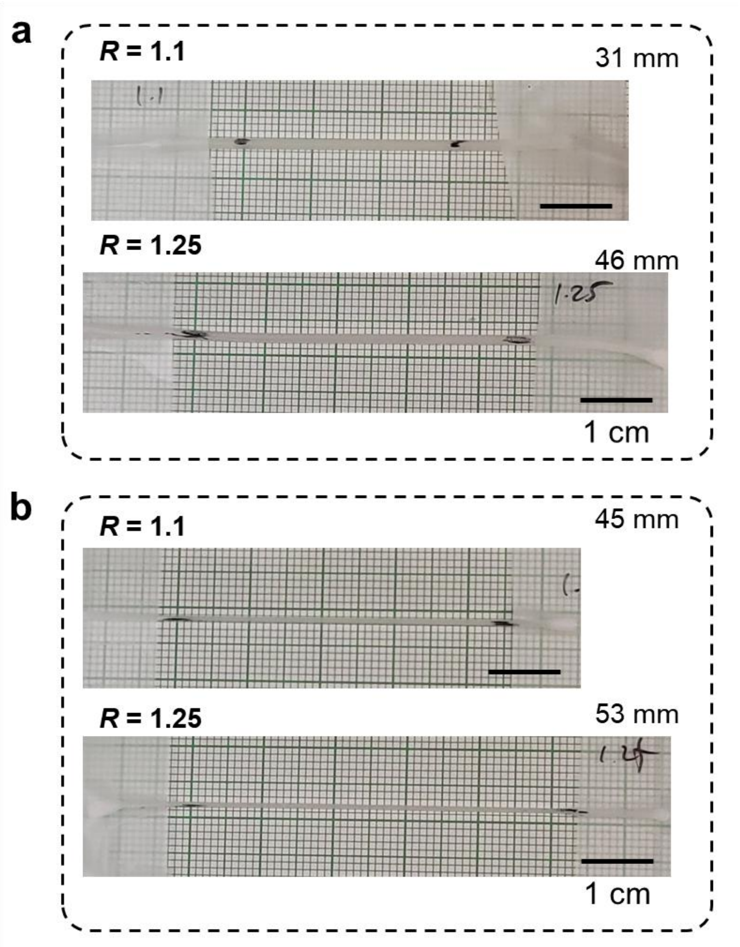

Figure S3. Kinetically trapped maximal strain of LCE fibers. (a) Photos of large diameter (LD) fibers with R = 1.1 (top) and R = 1.25 (bottom). (b) Photos of small diameter (SD) fibers with R = 1.1 (top) and R = 1.25 (bottom).

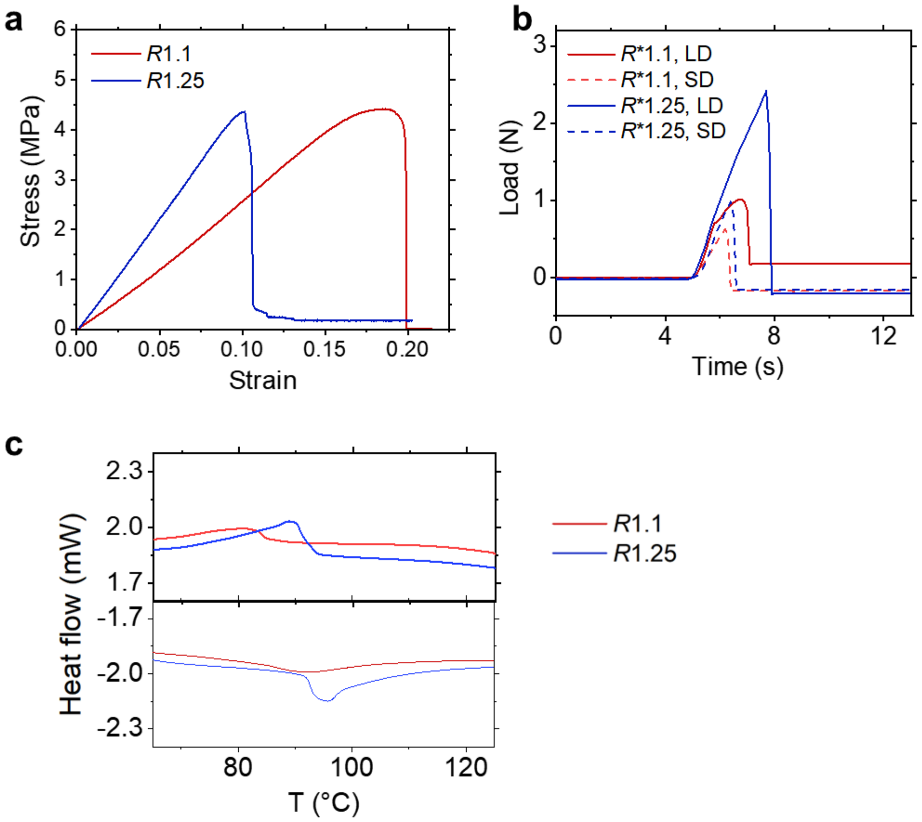

Figure S4. Mechanical behavior and thermal phase transition of the LCE fibers. (a) Tensile test on the stretched-only LCE fibers with $R = 1.1$ and 1.25. (b) Contraction force of stretched LCE fibers with different R and diameter. (c) Differential scanning calorimetry (DSC) data of the LCE fibers with $R = 1.1$ and 1.25.

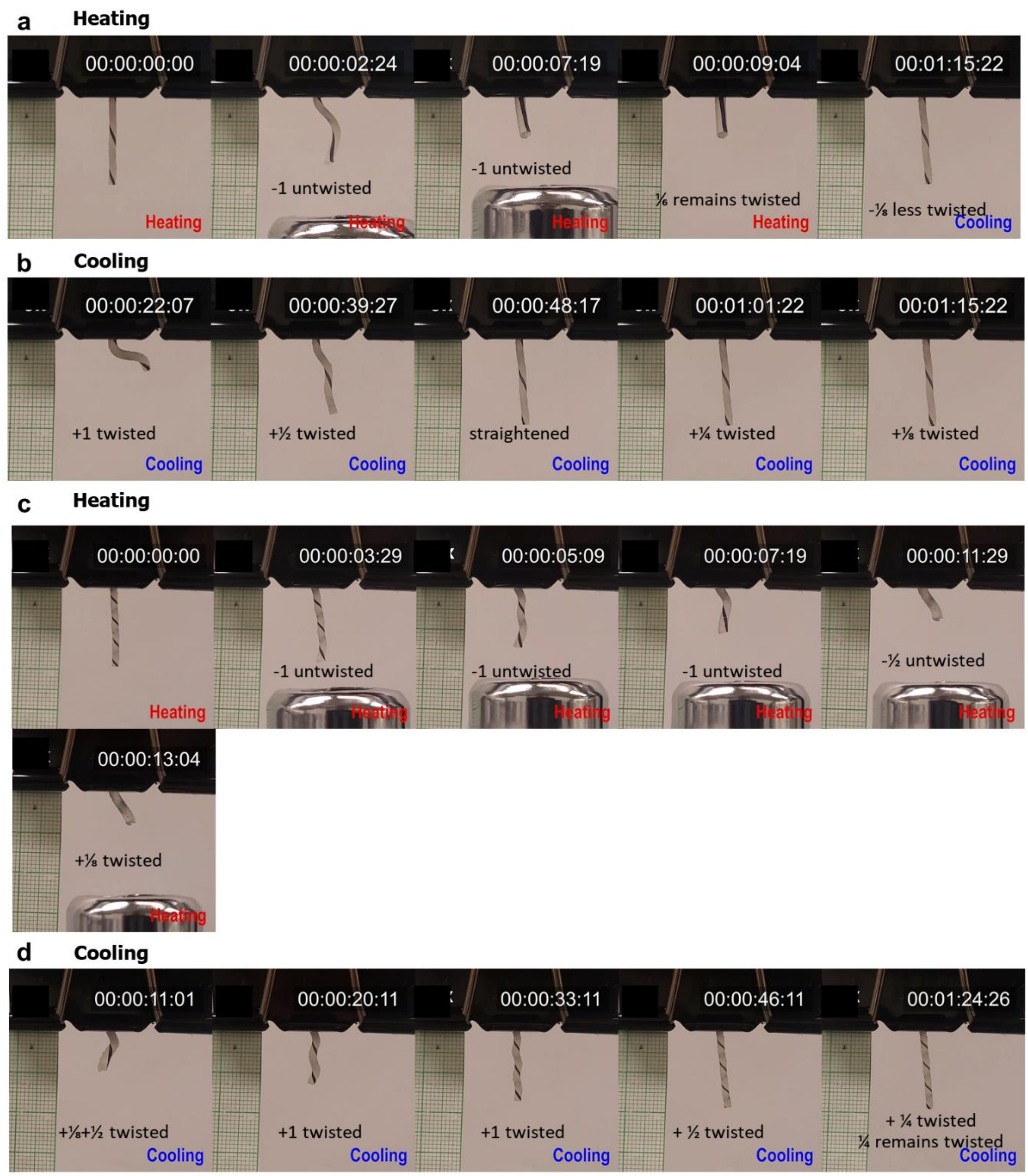

Figure S5. Torsional behavior of a twisted LCE fiber with a larger diameter. (a),(b) Deformation and recovery of an LCE fiber with $R = 1.1$ and a pitch of 10 mm, upon heating by a 220 °C heat gun and cooling at the room temperature, respectively. c,d) Same set as (a) and (b) with $R = 1.1$ and a pitch of 5 mm.

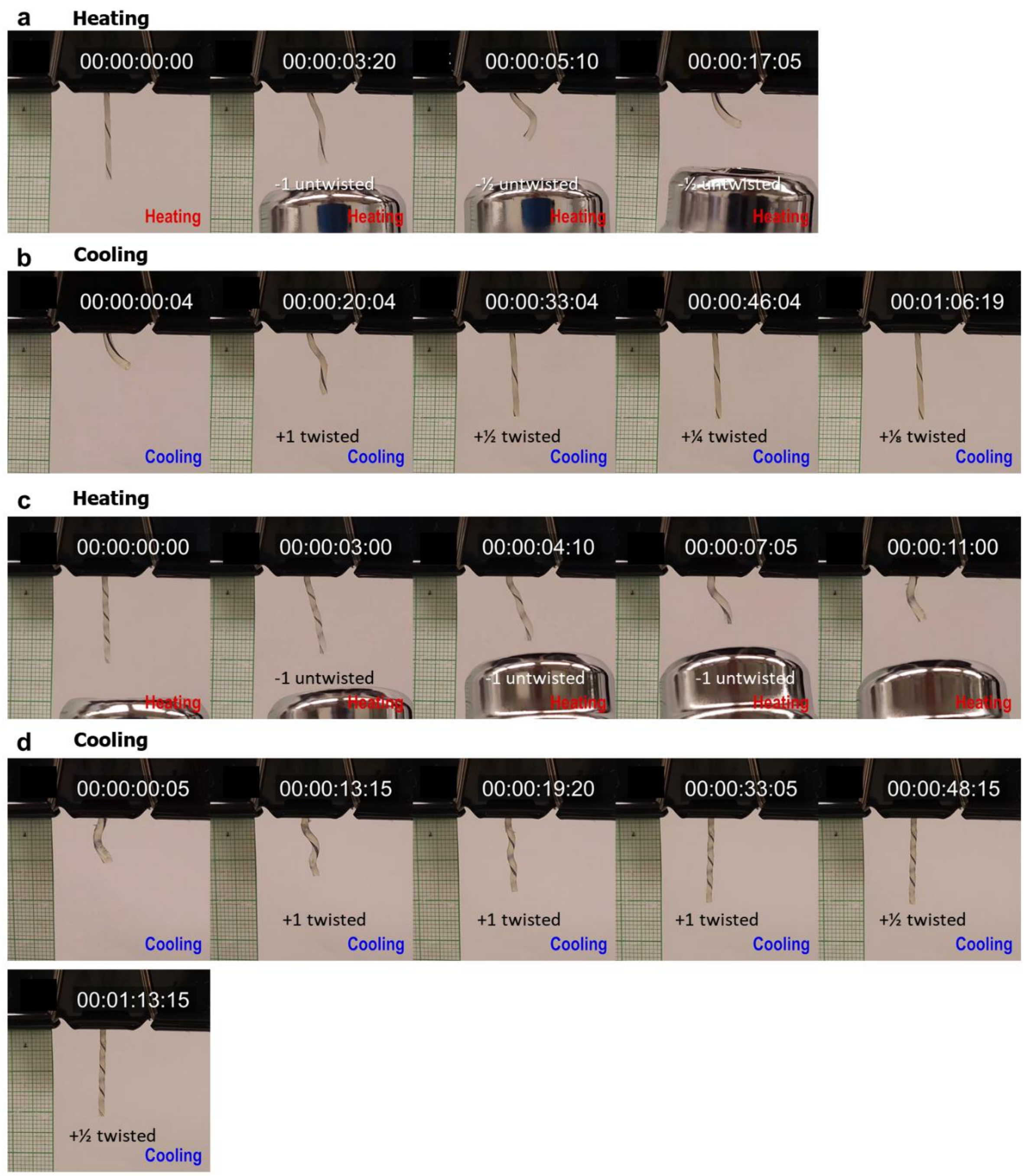

Figure S6. Torsional behavior of a twisted LCE fiber with an LD. (a),(b) Deformation and recovery of an LCE fiber with R = 1.25 and a pitch of 10 mm, upon heating by a 220 °C heat gun and cooling at the room temperature, respectively. c-d) Same set as (a) and (b) with R = 1.25 and a pitch of 5 mm.

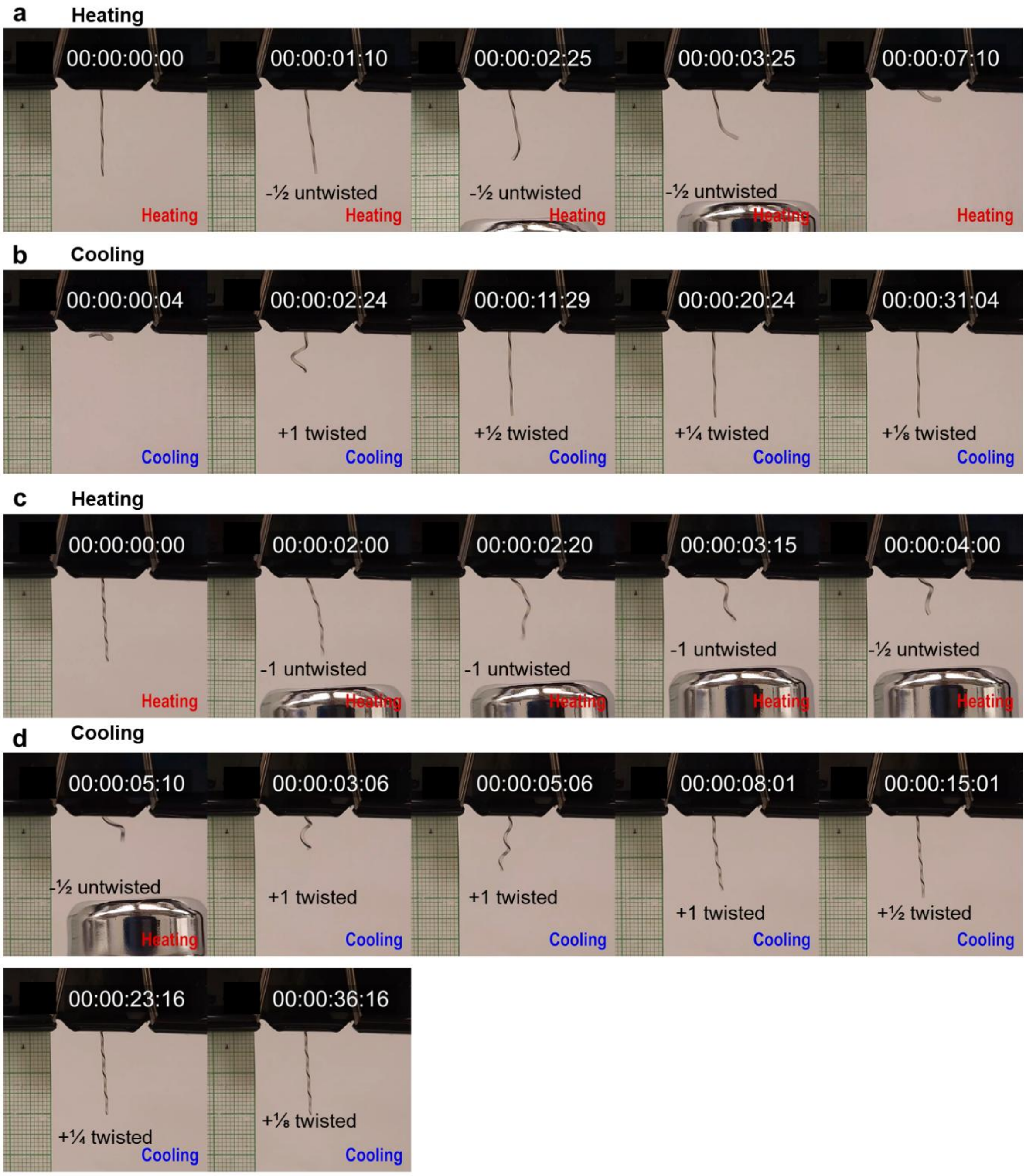

Figure S7. Torsional behavior of a twisted LCE fiber with an SD. (a),(b) Deformation and recovery of an LCE fiber with R = 1.25 and a pitch of 10 mm, upon heating by a 220 °C heat gun and cooling at room temperature, respectively. c**,**d) Same set as (a) and (b) with R = 1.25 and a pitch of 5 mm.

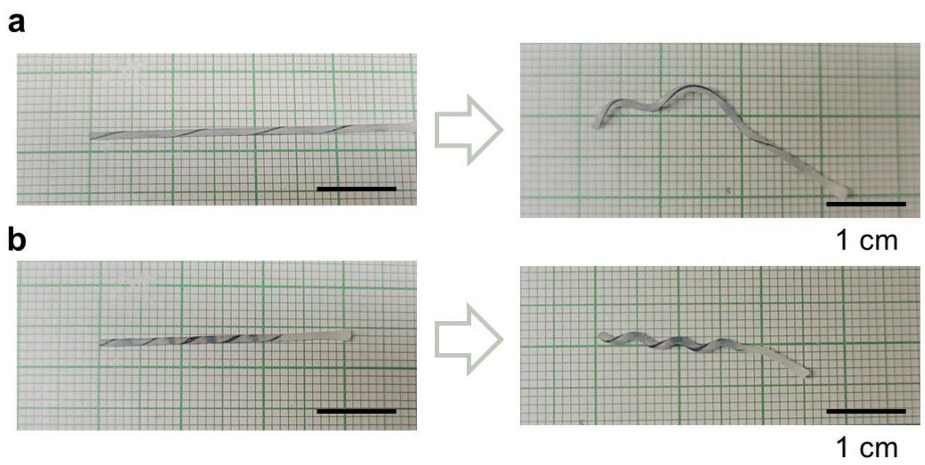

Figure S8. Incomplete recovery of an SD fiber of R = 1.1. (a),(b) Photos of the fiber with a pitch of 10 mm (a) and 5 mm (b), before (left) and after (right) the heating and cooling cycle.

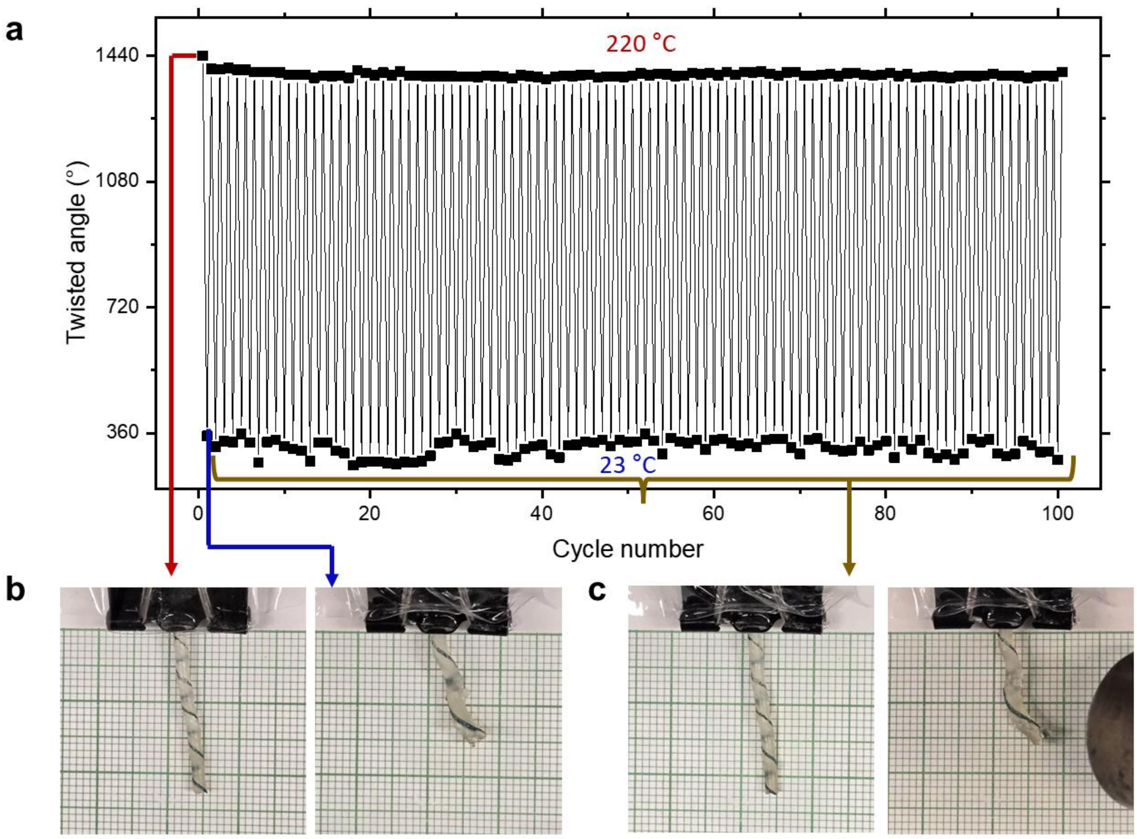

Figure S9. Torsional deformation-recovery cycles. (a) The change of the twisted angle in a 20-mm-long fiber over 100 cycles. (b-c) Digital photographs of the fiber before and after heating at 220 °C using a heat gun during the first (b) and a second (c) cycles.

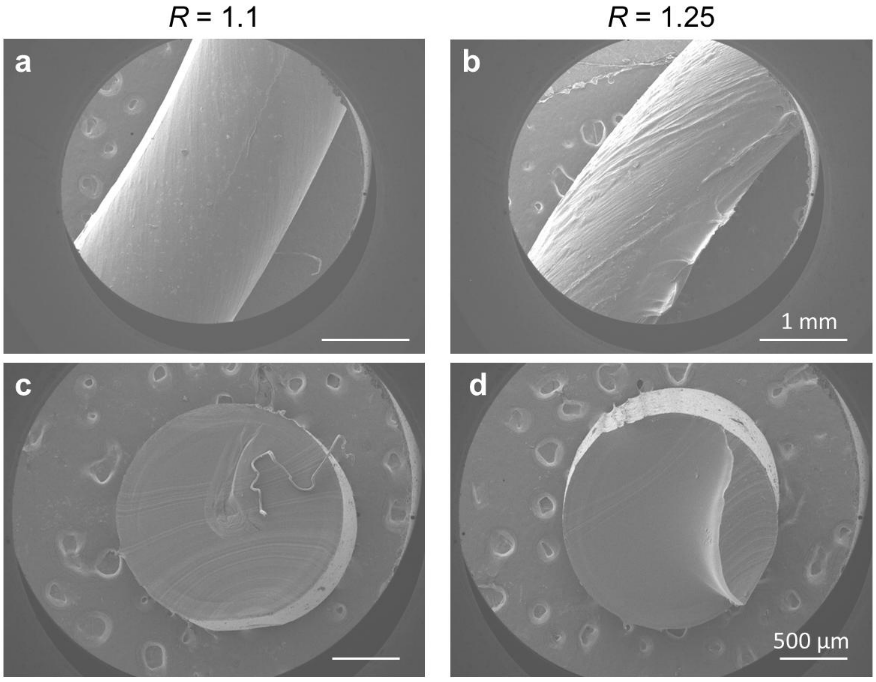

Figure S10. Non-porous LCE fibers. (a),(b) Scanning electron microscopy (SEM) images of the surface (a) and cross-section (b) of a twisted and unbent LD fiber of R = 1.1. c,d) Same set as (a) and (b) of a twisted and unbent LD fiber of R = 1.25.

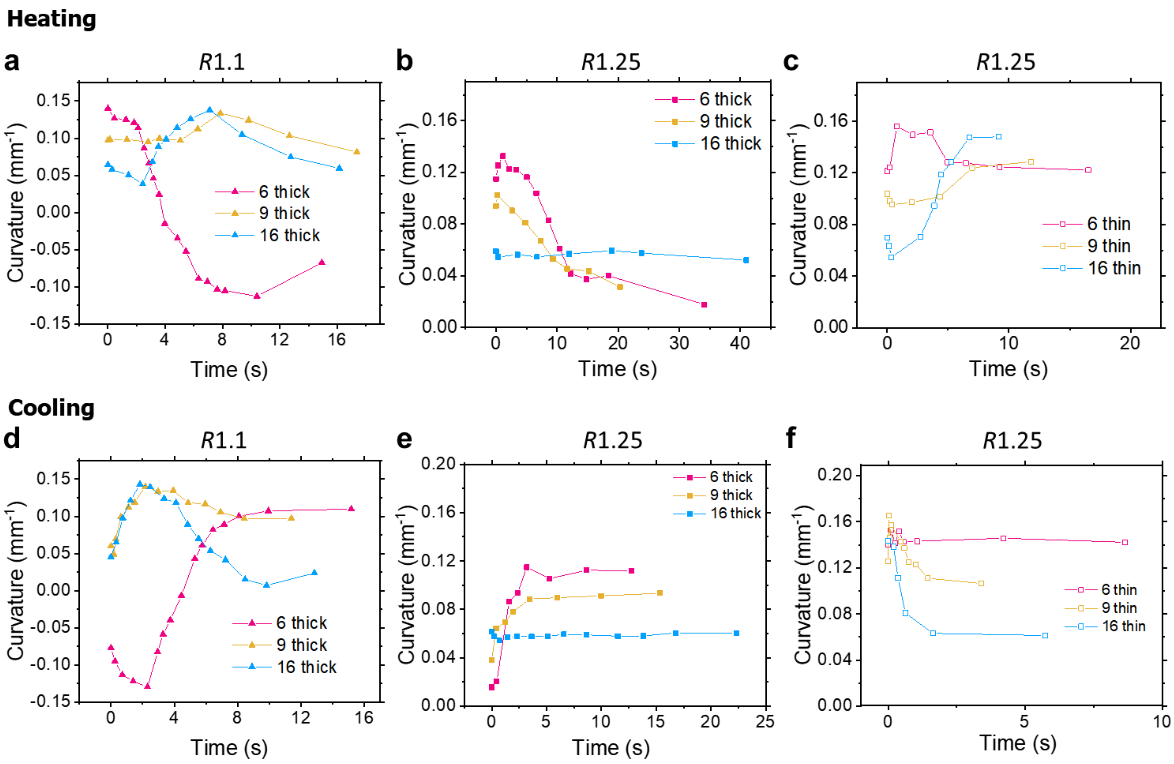

Figure S11. Curvature changes of the bent-only LCE fibers in a 140 °C silicone oil bath. (a–c) Time-dependent changes of the LD fibers of R = 1.1 (a) and R = 1.25 (b), and SD fibers of R = 1.25 (c). (d–f) The time-dependent recovery of the fibers corresponding to (a) to (c) upon cooling.

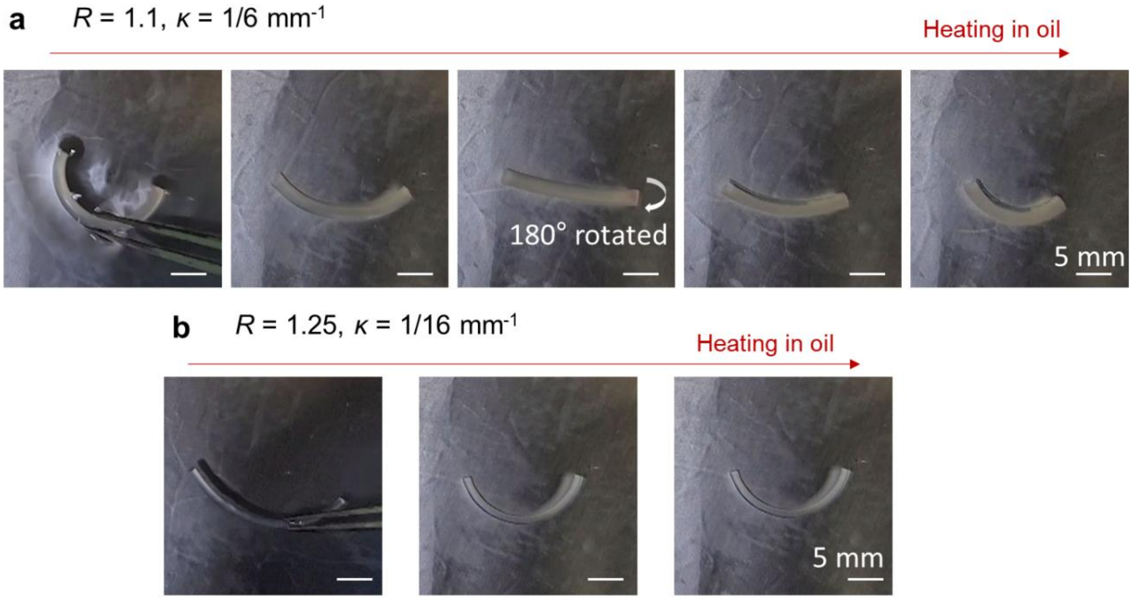

Figure S12. Anomalous flexural behavior of LCE fibers in 140 °C silicone oil. (a),(b) Time-lapse images of the LD fiber with R = 1.1 and κ = 1/6 mm^{-1} (a) and the SD fiber with R = 1.25 and κ = 1/16 mm^{-1} (b).

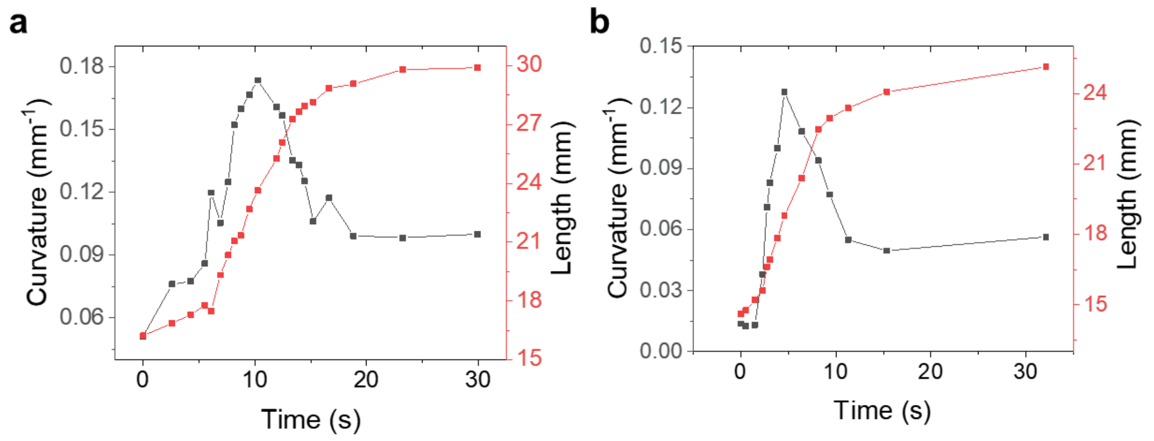

Figure S13. Curvature and length changes of LCE fibers upon cooling in a room-temperature oil bath after heating in a 140 °C oil bath. (a),(b) SD fibers with R = 1.25 and κ of 1/9 (a) and 1/16 mm^{-1} (b), respectively.

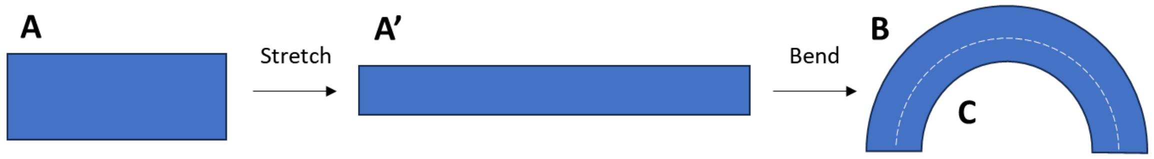

Figure S14. Illustrations of a stretched and bent LCE fiber.

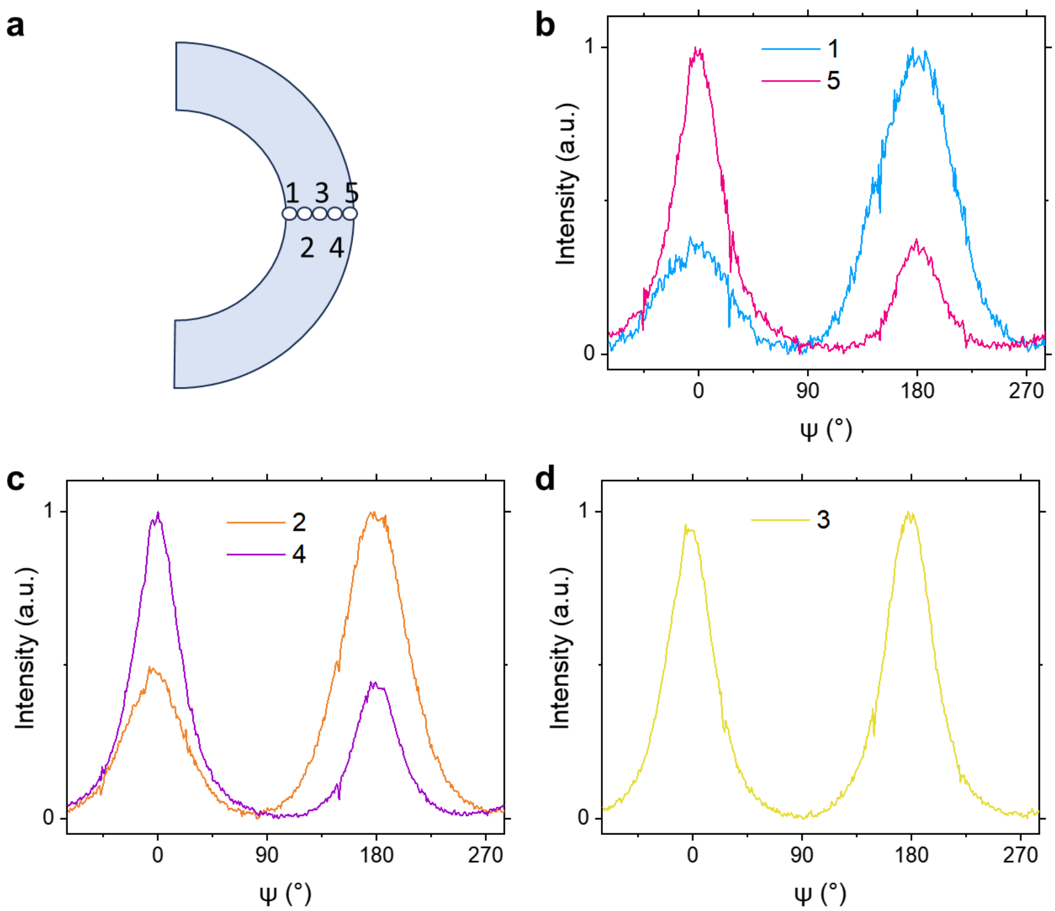

Figure S15. Gradual change of the degree of alignment in an *R*1.1 bent fiber with *κ* of 6 mm^{-1}. (a) Illustration indicating the spots measured by X-ray diffraction. (b–d) Azimuthal scan from the spots 1 and 5 (b), the spots 2 and 4 (c), and the spot 3 marked in (a).

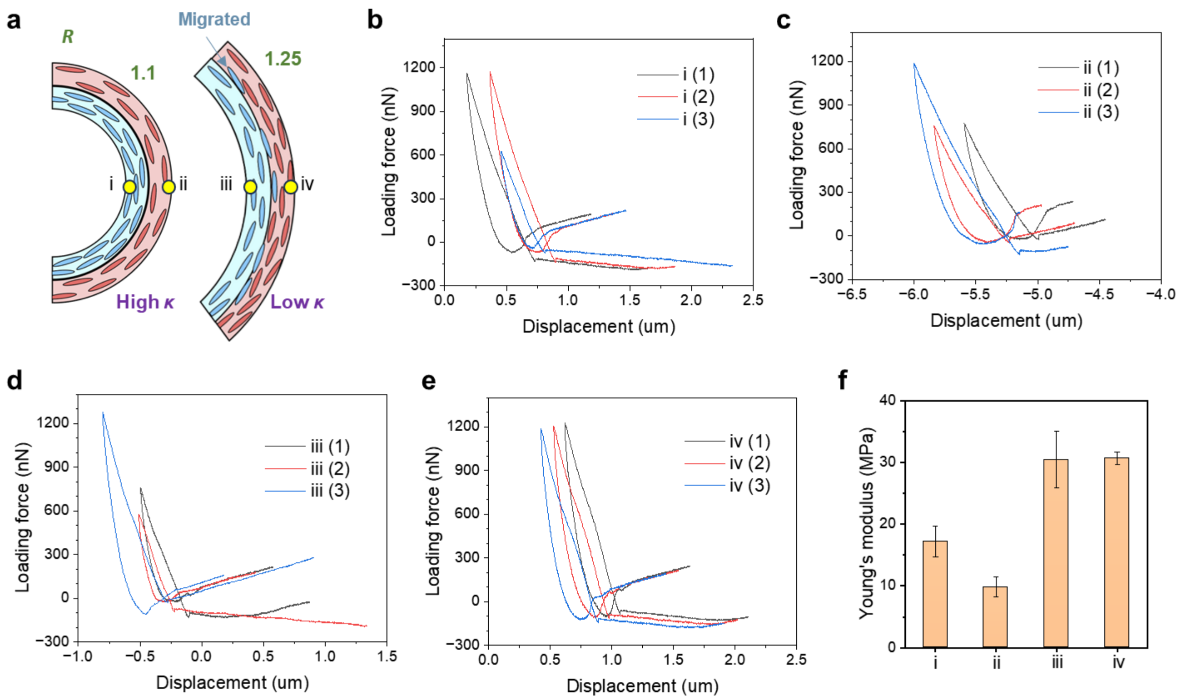

Figure S16. Atomic force microscopy (AFM) indentation test on the cross-section of the bent fibers. (a) Illustration indicating the spots measured at the cross-section by AFM. (b–e) Force-displacement curves from the measurement at the spot (i) (b), spot (ii) (c), spot (iii) (d), and spot (iv) (e). (f) Comparison of Young's modulus at different spots.

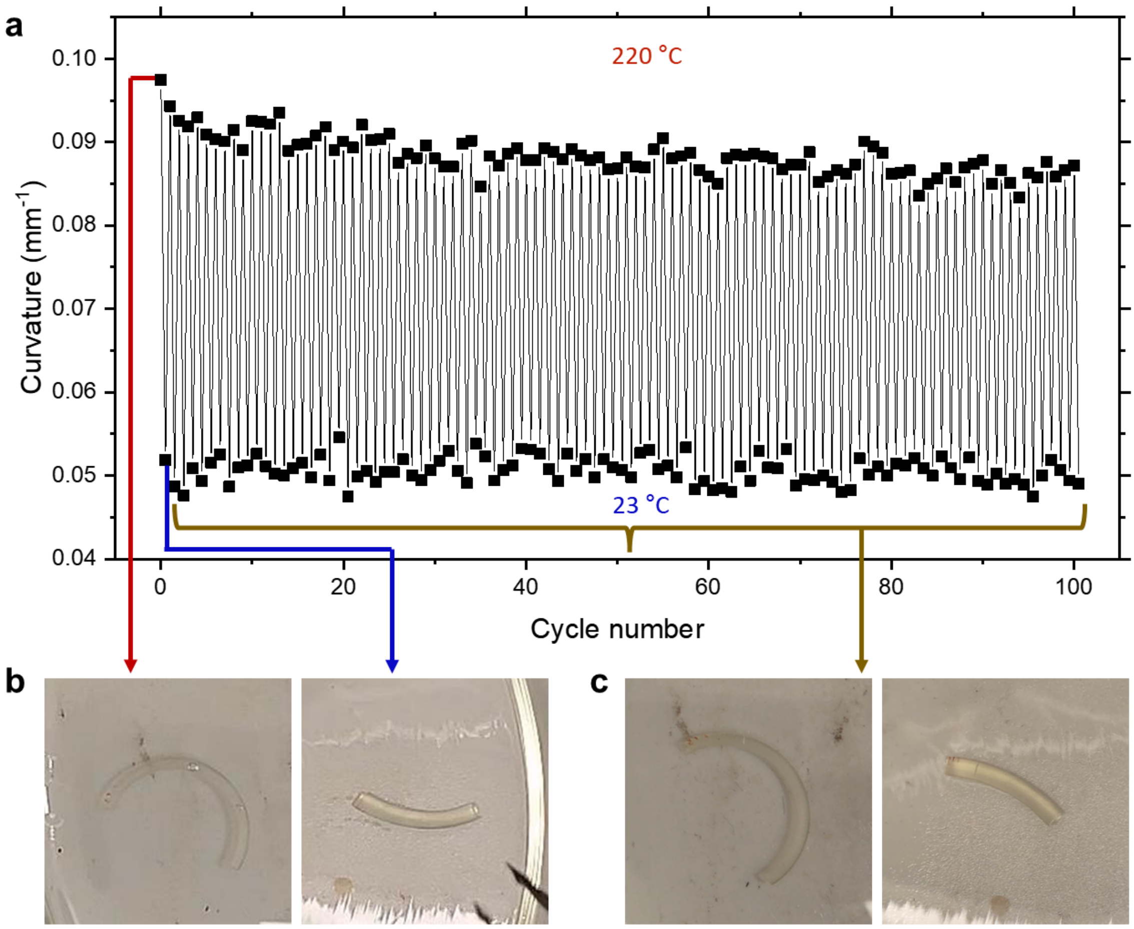

Figure S17. Flexural deformation-recovery cycles. (a) The change of curvatures of a bent fiber over 100 cycles. (b-c) Digital photographs before and after heating in a 220 °C oil during the first (b) and second (c) cycles.

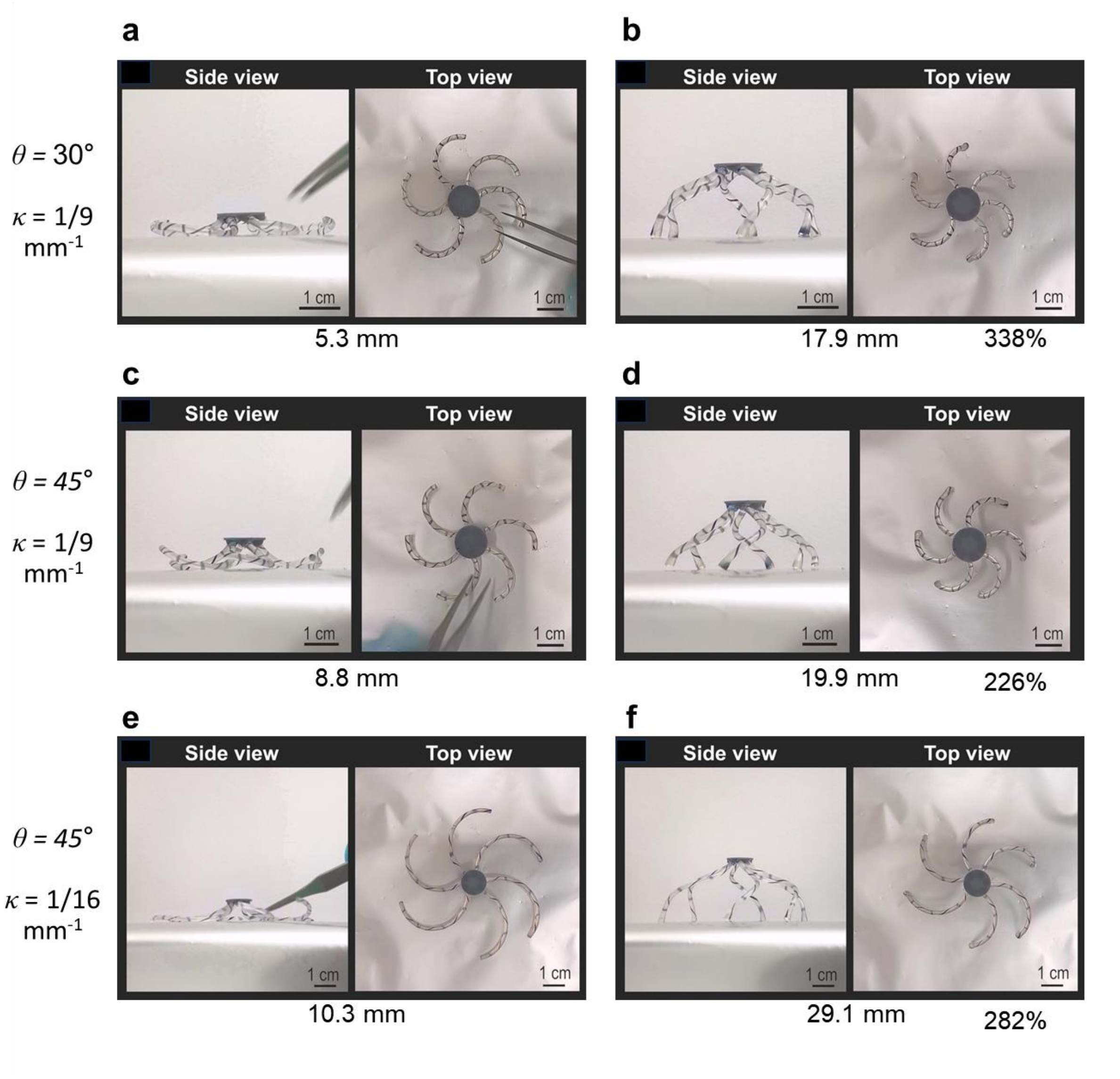

Figure S18. Customized lifting LCE robots on a 180 °C hot surface. (a),(b) Side- (a) and top- (b) views of the robot, where LD LCE fibers have parameters of (R, # of twists per half loop, $1/\kappa$) = (1.1, 4, 9) and θ = 30°. (c),(d) Same set as (a) and (b) when θ = 45°. (e),(f) Same set as (a) and (b) when the parameters are (1.1, 4, 16) and θ =45°.

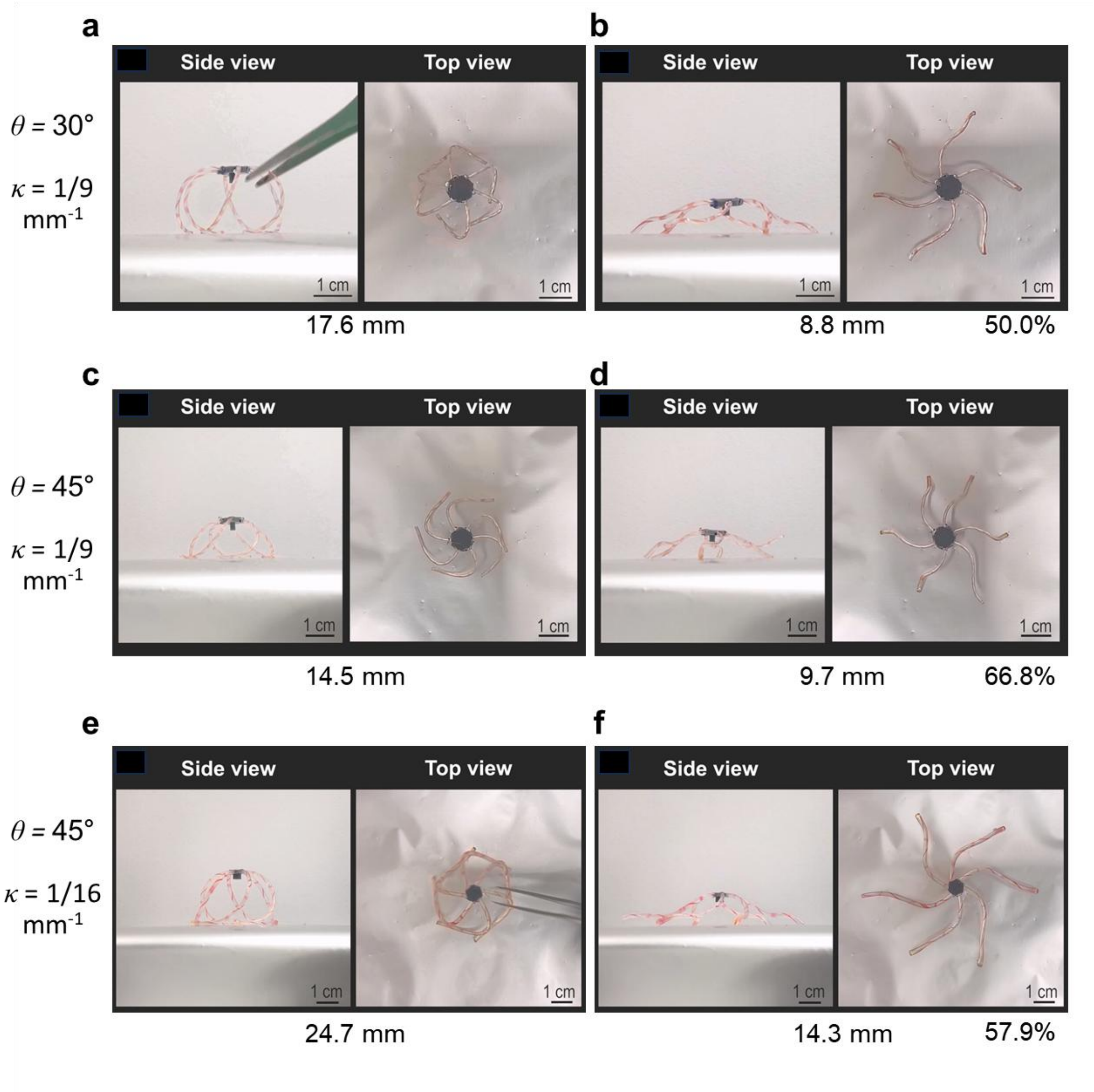

Figure S19. Customized lowering LCE robots on a 180 °C hot surface. (a),(b) Side (a) and top (b) views of the robot, where SD LCE fibers have parameters of (R, # of twists per half loop, $1/\kappa$) = (1.25, 3, 9) and θ, defined in Figure 3B(i), is 30°. (c),(d) Same set as (a) and (b) when θ is 45°. (e),(f) Same set as (a) and (b) when the fibers have an LD, parameters are (1.1, 4, 16), and θ is 45°.

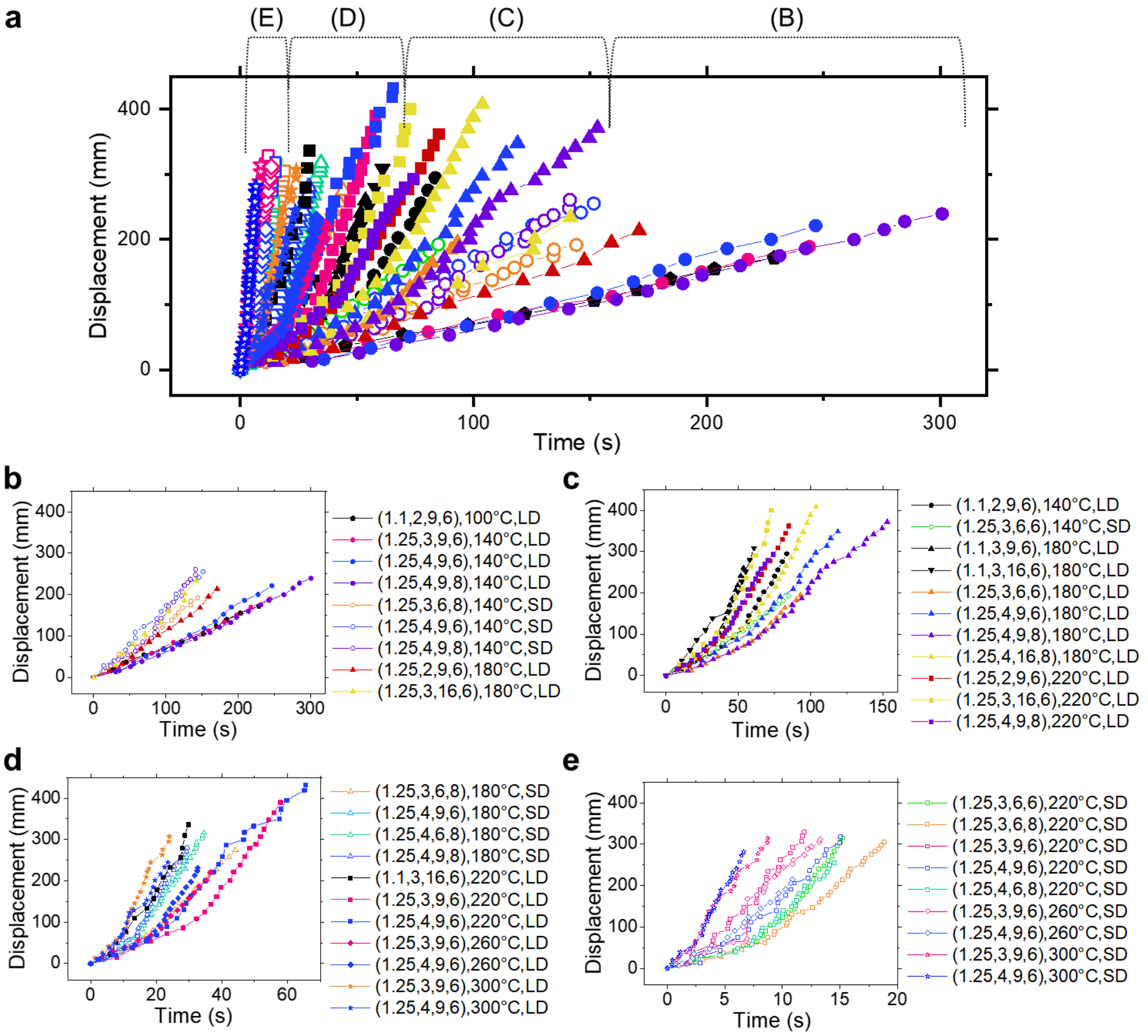

Figure S20. Speed analysis of various rollbots. (a) Displacement vs. time plots for all tested rollbots. (b–e) Magnified views of the data marked in (a).

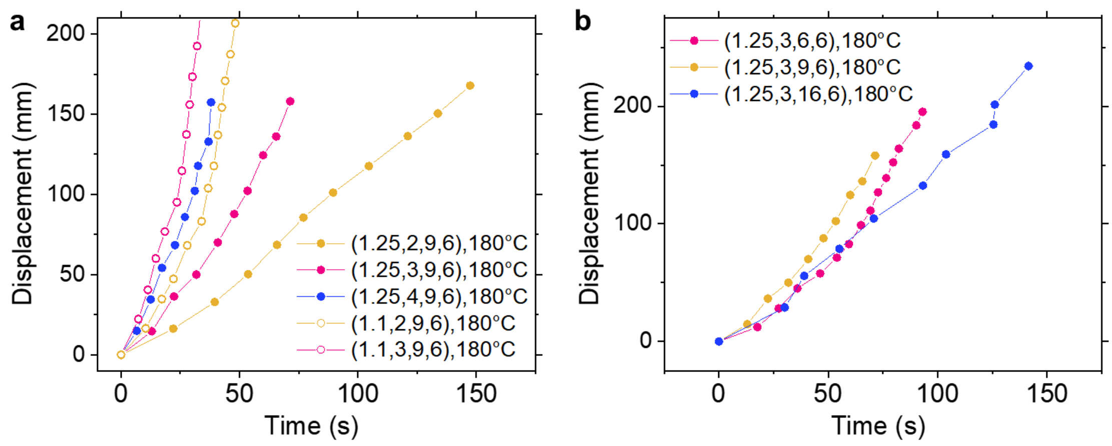

Figure S21. Effect of twist density of fibers and curvature on the rollbots' speed. (a),(b) Displacement vs. time plot for rollbots with LD fibers of varying twist density (a) and curvature (b).

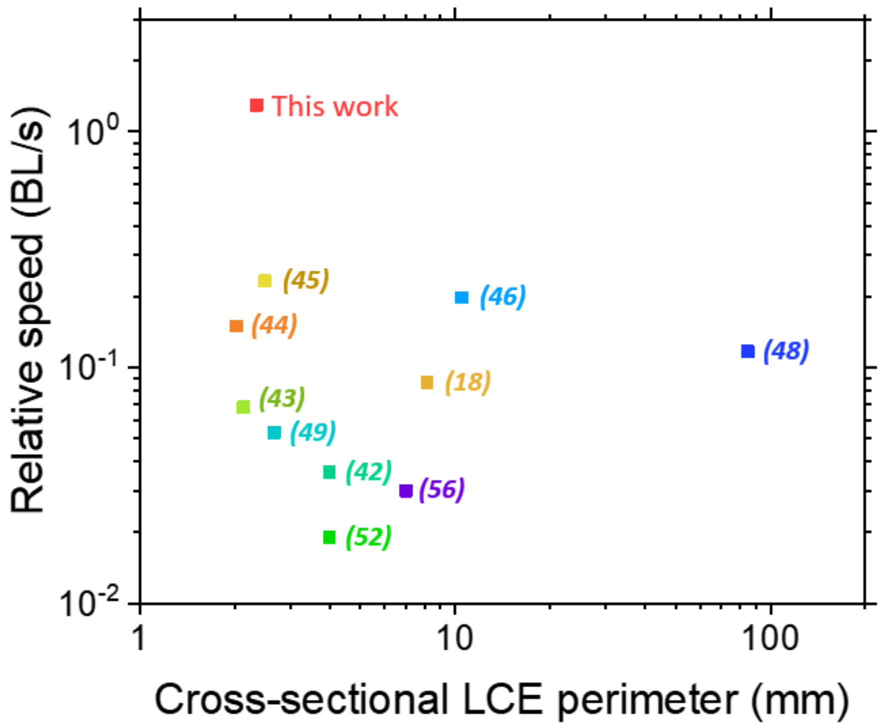

Figure S22. Ashby plot showing the relative speed (BL/s) of LCE soft robots as a function of their cross-sectional perimeter. The body length (BL) is defined as the longer dimension of the rollbot parallel to the rotation axis.

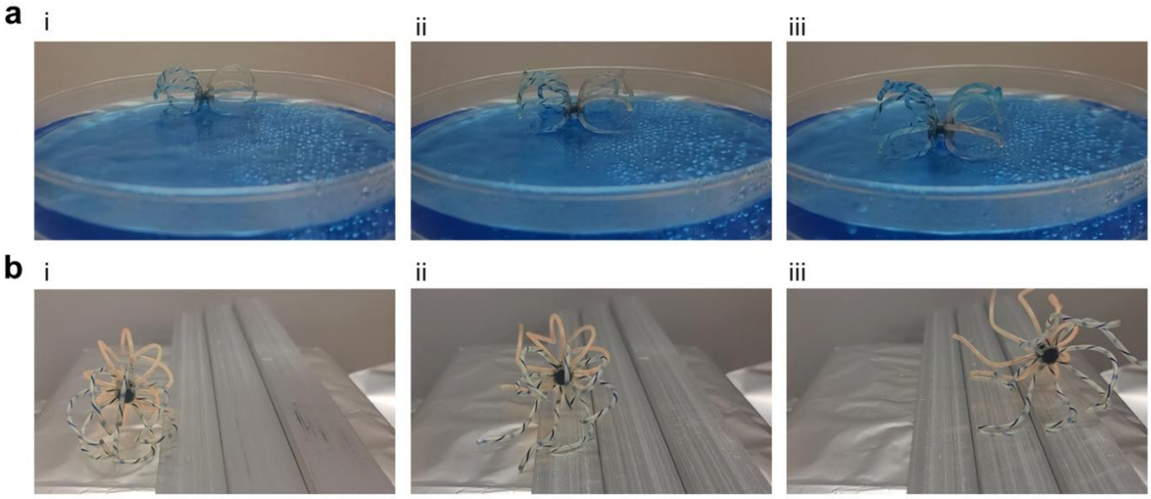

Figure S23. Locomotion of rollbots in unusual environments. (a) Rolling locomotion of the (1.25. 4, 9, 6) rollbot in shallow water at 95 °C. (b) Climbing locomotion of the (1.1, 4, 16, 8) rollbot on stairs with 6-mm steps.

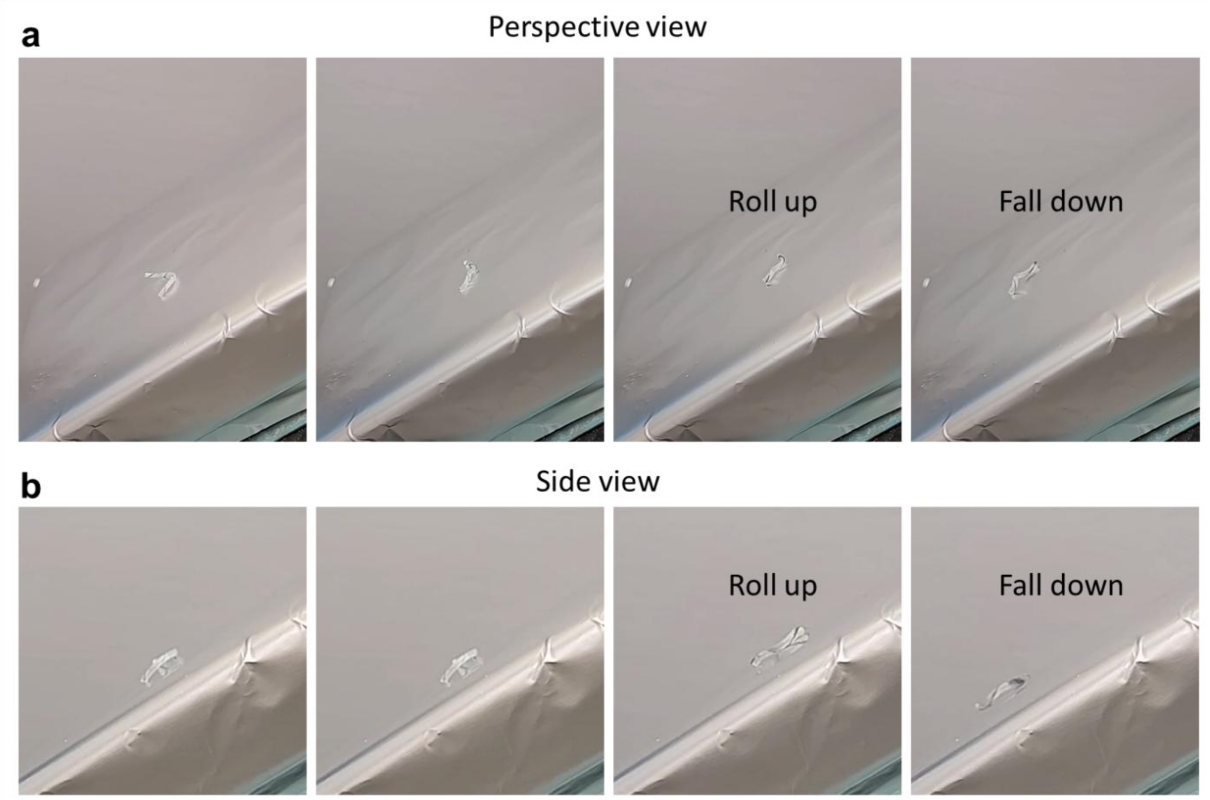

Figure S24. Photos of a twisted and bent fiber of (1.25, 4, 9), climbing on a 32.5° ramp on a 180 °C hot plate. (a) Perspective view. (b) Side view.

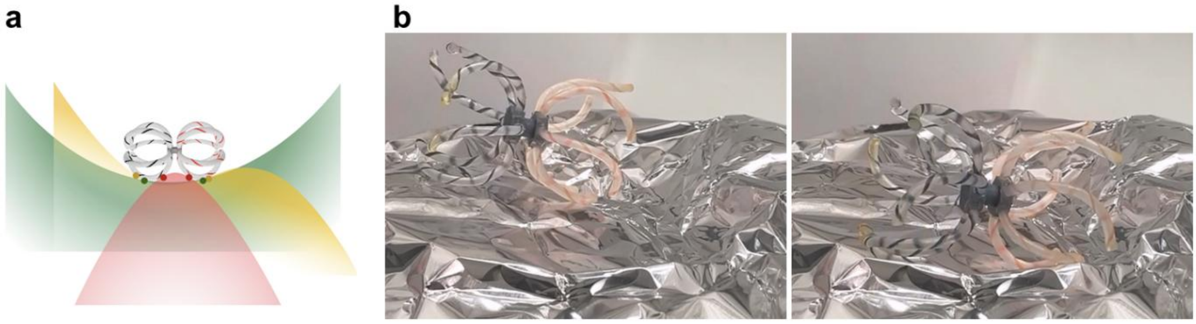

Figure S25. Locomotion of a rollbot on a random terrain heated on a 220 °C surface. (a) Illustration of a rollbot smaller than that demonstrated in Figure 6D. The fibers of the rollbot have point contacts at any random terrain due to their curved geometry. (b) Time-lapse photo of the rolling rollbot of (1.1, 4, 9, 6) on a randomly crumpled terrain.

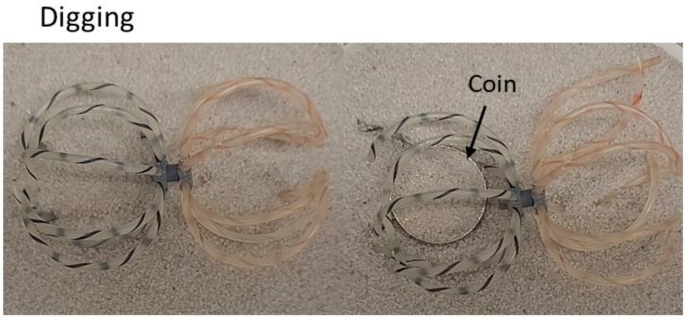

Figure S26. Digging for a U.S. quarter dollar coin buried in sand heated on a 220 °C surface by the (1.1, 4, 16, 6) rollbot.

Movie legends

Movie S1. Out-of-plane deformation of half-arc *R*1.25 twisted 4 times per half loop on a 180 °C surface.

Movie S2. Deformation and recovery of twisted LCE fibers by a 220 °C heat gun.

Movie S3. Deformation and recovery of bent LCE fibers by a 220 °C heat gun.

Movie S4. Anomalous flexural deformation of *R*1.1 with κ of 1/6 mm^{-1} in 140 °C oil.

Movie S5. Anomalous flexural deformation of *R*1.25 with κ of 1/16 mm^{-1} in 140 °C oil.

Movie S6. Octopus-inspired robot with a lifting motion on a 180 °C surface.

Movie S7. Octopus-inspired robot with a lowering motion on a 180 °C surface.

Movie S8. Octopus-inspired robot with a rotating motion on a 180 °C surface.

Movie S9. Octopus-inspired robot with a tilting motion on a 180 °C surface.

Movie S10. Rolling locomotion of a (1.25, 4, 9, 6) rollbot on a 180 °C surface.

Movie S11. Spinning locomotion of a (1.25, 4, 9, 8) rollbot on a 180 °C surface.

Movie S12. (1.25, 4, 16, 8) rollbot overcoming water's surface tension in 95 °C water.

Movie S13. (1.25, 4, 16, 8) rollbot climbing a 32.5° ramp on a 180 °C surface.

Movie S14. Cargo-dragging rollbots on a 180 °C surface.

Movie S15. Rollbots' locomotion on a randomly crumpled terrain on a 220 °C surface.
